# Nitric-Oxide Synthase trafficking inducer (NOSTRIN) is an emerging negative regulator of colon cancer progression

**DOI:** 10.1186/s12885-022-09670-6

**Published:** 2022-05-31

**Authors:** Madhurima Paul, Tamal Kanti Gope, Priyanka Das, Rupasri Ain

**Affiliations:** Division of Cell Biology and Physiology, CSIR-Indian Institute of Chemical Biology, 4, Raja S.C. Mullick Road, Jadavpur, Kolkata, West Bengal 700032 India

**Keywords:** EMT, Cancer stem cell, Colonosphere, Colorectal cancer, Prognostic marker, HCT116

## Abstract

**Background:**

NOSTRIN, abundantly expressed in colon, was reported to be anti-angiogenic, anti-invasive and anti-inflammatory. NOSTRIN expression was inversely related to survival of pancreatic ductal adeno-carcinoma patients. Yet its function and regulatory mechanism in CRC remains elusive.

**Methods:**

NOSTRIN’s influence on EMT of CRC cells were analysed using realtime PCR array containing the functional EMT-transcriptome followed by western blotting. Regulation of oncogenic potential of CRC cells by NOSTRIN was elucidated using soft agar colony formation, trans-well invasion, wound healing and colonosphere formation assays. Biochemical assays were used to reveal mechanism of NOSTRIN function. Human CRC tissue array was used to test NOSTRIN mark in control and CRC disease stages.

**Results:**

We showed here that CRC cell lines with less NOSTRIN expression has more invasive and migratory potential. NOSTRIN affected EMT-associated transcriptome of CRC cells by down regulating 33 genes that were functionally annotated to transcription factors, genes important for cell growth, proliferation, migration, cell adhesion and cytoskeleton regulators in CRC cells. NOSTRIN over-expression significantly reduced soft agar colony formation, wound healing and cell invasion. In line with this, RNA interference of *Nostrin* enhanced metastatic potential of CRC cells. Furthermore, stable overexpression of NOSTRIN in CRC cell line not only curtailed its ability to form colonosphere but also decreased expression of stemness markers CD133, CD44 and EpCAM. NOSTRIN’s role in inhibiting self-renewal was further confirmed using BrdU incorporation assay. Interestingly, NOSTRIN formed immune-complex with Cdk1 in CRC cells and aided in increase of inhibitory Y15 and T14 phosphorylation of Cdk1 that halts cytokinesis. These *ex vivo* findings were substantiated using human colon cancer tissue array containing cDNAs from patients’ samples with various stages of disease progression. Significant decrease in NOSTRIN expression was found with initiation and progression of advanced colon cancer disease stages.

**Conclusion:**

We illustrate function of a novel molecule, NOSTRIN in curtailing EMT and maintenance of CRC cell stemness. Our data validates importance of NOSTRIN mark during onset and disease progression of CRC indicating its diagnostic potential.

**Supplementary Information:**

The online version contains supplementary material available at 10.1186/s12885-022-09670-6.

## Background

A highly heterogeneous and the third most prevalent disease, colorectal cancer (CRC) is globally the fourth leading cause of deaths related to cancer [1, 2]. Reprogramming of gene expression that drives cancer stem cell self-renewal and turns on metastatic progression enables CRC disease progression. Several molecular pathways and genetic aberrations have already been identified that contribute to development and metastatic progression of this disease, yet overall survival rate of colorectal cancer patients seem to be low [3, 4]. Thus, further identification of new targets to restrict colon cancer progression as well as understanding the underlying regulatory mechanisms that drive metastatic progression and CRC stem cell self-renewal is a crucial step towards improving patient outcomes.

NOSTRIN (Nitric Oxide Synthase Trafficking INducer) is abundantly expressed in colon. It is classically known as an endothelial cell protein that sequesters eNOS and inhibits NO production [5]. However, various eNOS dependent and independent functions of NOSTRIN have been demonstrated till date [6–9]. Owing to its function as an adapter protein, NOSTRIN interacts with other proteins depending on cellular context and exhibits pleiotropic functions [10–12]. Interestingly, NOSTRIN has been shown to possess potent anti-angiogenic, anti-invasive and anti-inflammatory functions in endothelial cells [9]. Besides, NOSTRIN was shown to be involved in inhibition of cell cycle progression [12]. Angiogenesis and invasion form an integral mechanism of cancer growth and metastasis [13], and aberrant cell cycle progression is a hallmark of cancer progression. We, therefore, argued that NOSTRIN might possess anti-cancer properties in CRC cells.

Interestingly, NOSTRIN was identified as one of the 36 gene signatures representing prognostic predictor of clinical outcome in pancreatic ductal adenocarcinoma (PDAC), wherein NOSTRIN down-regulation was associated with poor outcome [14]. Similar results in PDAC patients were also reported by Wang et al. [15]. In addition, overexpression of NOSTRIN curtailed cell invasion and gemcitabine-induced apoptotic cell death in human pancreatic cancer cell line [15]. NOSTRIN’s ability to sequester eNOS and consequently its dampening effect on NO production by endothelial cells has been implicated in cancer progression [16]. Interestingly, combined use of NOS inhibitors with 5-fluorouracil showed enhanced inhibition of cell proliferation and migration in CRC cell lines [17]. However, there are no reports directly linking NOSTRIN with colorectal cancer. Owing to its abundance in colon and its known functions, we sought to investigate the role of NOSTRIN in regulating colorectal cancer.

In this study, we elucidated the role of NOSTRIN using two CRC cell lines followed by evaluation of NOSTRIN expression in progressive disease stages of CRC. Two CRC cell types used in our study reflect molecular alterations and pharmacogenomics of primary tumors [18–20]. The cell lines belonged to either of the two distinct subgroups: a) *colon-like* (HT29 cells) and b) *undifferentiated* (HCT116) based on their differential expression in DNA, RNA and protein levels [21]. HT29 cell line represent *colon-like* cell types and are characterized by higher expression of gastro-intestinal marker genes including those that represses EMT program and showing more differentiated property. On contrary, HCT116 cell line represents undifferentiated subtype and is known to be more aggressive and poorly differentiated showing up-regulated EMT signature. Clinically the *undifferentiated* subtype is associated with poor prognosis and represents advanced stages of the disease.

The findings illustrated in this study have broad biological implications, as enhanced NOSTRIN expression leading to compromised EMT and decreased stemness of cancer cells might inhibit tumorigenesis and colorectal cancer progression. Furthermore, diminished NOSTRIN mark with CRC disease progression put forth NOSTRIN’s potential to be used as a prognostic marker for CRC.

## Methods

### Cell culture

Human colorectal cancer cell lines, COLO 205, SW480, HCT116, HT29 were obtained from American Type Culture Collection (USA). HCT116 and HT29 cells were grown in McCoy’s 5a Medium (Sigma Aldrich, USA); COLO 205 was grown in RPMI-1640 (Invitrogen, USA) and SW480 in Lebovitz’s L-15 medium (Sigma Aldrich, USA) supplemented with 10% Fetal Bovine Serum (Invitrogen, USA) 1% Penicillin-Streptomycin (Invitrogen, USA) and 1% Glutamax (Invitrogen, USA). These cells were maintained in presence of 5% CO_2_ at 37 °C in a humidified incubator.

### Cloning, characterization of human NOSTRIN cDNA and generation of stable cell line

RNA was isolated from human term placenta; reverse transcribed using M-MLV Reverse Transcription kit (Invitrogen, USA). Human term placental tissue sample was obtained from Calcutta National Medical College & Hospital (CNMC), post-delivery. Full length human *Nostrin* (NM_001171631.1) cDNA was amplified from the cDNA pool using LA-Taq DNA polymerase (TaKaRa, Clontech, USA). Primers used for full length NOSTRIN were Fwd: 5′-AATTAAGCTTATGAGGGACCCACTGACAG-3′ and Rev.: 5′-TAATGGATCCTTATGCCTTTGTAGCTGTG-3′. The forward primer contained Hind III and reverse primers contained BamH1 restriction sites. The amplified cDNAs were cloned using p3XFLAG-CMV-10 (catalogue No.# E7658, Sigma Aldrich, USA) vector following transformation in Mach1™-T1^R^ (Sigma Aldrich, USA) cells. Four clones were restriction digested and confirmed for correct size. Correctness of the sequence was confirmed by Sanger dideoxy sequencing.

HCT116 cells were transfected with either *Nostrin* cDNA in p3XFLAG-CMV-10 or the empty vector backbone using Lipofectamine (LTX) and Plus reagent (Invitrogen, USA). Transfected cells were grown for 48 h and were then maintained in 500 μg/ml G418 (Invitrogen, USA) containing complete growth medium for HCT116 cells to select cells with stable *Nostrin* cDNA integration followed by cloning of cells using serial dilution method. Approximately 7–8 clones were then evaluated for *Nostrin* over-expression by quantitative real time PCR. These clones were then tested for overexpression of NOSTRIN protein and one of the highest expressing clones were then selected for further studies. The selected positive cell clone was cultured for approximately 2 months in 300μg/ml G418 containing medium.

#### Transfection and reagents

NOSTRIN knockdown in HT29 colon cancer cells were done by transfecting the cells with two pre-validated silencer select siRNAs targeting the coding region of human NOSTRIN (Assay ID: s41846 and s41847, Ambion, USA) using Lipofectamine RNAiMax reagent (Invitrogen, USA). Cells treated with scramble siRNA were used as control. siRNA treatment was done in a dose dependent manner and a concentration of 50 nM and 100 nM were used. Down-regulation was assessed in transcript level using quantitative real time PCR and in protein level by western blotting. Further knockdown experiments were performed using the concentration showing maximum down-regulation.

#### RNA isolation & quantitative real-time PCR analysis

Total RNA was isolated from cells using TRIZOL reagent (Invitrogen, USA) as per manufacturer’s protocol. Extracted RNA was reverse transcribed using the M-MLV Reverse Transcription kit (Invitrogen, USA). Quantitative-real time PCR reaction was set in a 7500 real- time PCR system (Applied Biosystems, USA) with a 10-fold dilution of cDNA and Power SYBR GREEN PCR Master Mix (Applied Biosystems, USA) using standard PCR conditions as described previously [9]. Primers used in this study have been listed in Table 1. *Rpl7* was used as an endogenous control for normalisation of the gene of interest.

**Table 1:** Primer sequences used for qRT-PCR analysis

Sl. No.	Primer name		Sequence (5’ to 3’)	Gene Bank Accession no.
1	*Vim*	Fwd	CCAGATTCAGGAACAGCATGTC	NM_003380
		Rev	TCAGCAAACTTGGATTTGTACCA	
2	*Msn*	Fwd	AAGCCCCGGACTTCGTCTT	NM_002444
		Rev	TTCATCTGCTGCACCTCAATG	
3	*Stat3*	Fwd	AGTGACTGGTTGTTTCCATTCAGATC	NM_003150
		Rev	GAGCAGCACCTTCAGGATGTC	
4	*Smad2*	Fwd	GAAATGCCACGGTAGAAATGACA	NM_005901
		Rev	TAGGGTGCCAGCCATATCTC	
5	*Col3A1*	Fwd	CGGAAATGATGGTGCTCCTG	NM_000090
		Rev	TGTCTCCTTTGTCACCACCA	
6	*F11r*	Fwd	GACACCACCAGACTCGTTTGC	NM_016946
		Rev	GTTGCCGCCTTCCTCAGA	
7	*Itgα5*	Fwd	CTCAGGAACGAGTCAGAATTTCG	NM_002205
		Rev	GTCCTCTATCCGGCTCTTGCT	
8	*Vcan*	Fwd	GCGGGATTGAAGACACACAAGA	NM_004385
		Rev	CGCTCTGGAGTTGCTATGACTG	
9	*Nostrin*	Fwd	CAGCACCTCCTCCTTCTCTGA	NM_001171631
		Rev	TGGCTGGGTTGAGGTCTTTG	
10	*Rpl7*	Fwd	AATGGCGAGGATGGCAAGA	NM_001363737
		Rev	AAGGCGAAGAAGCTGCAACA	

### Quantitative RT^2^ Profiler PCR Array

A quantitative real-time PCR-based human EMT RT^2^ Profiler PCR array (catalogue No. 330231 PAHS-090ZA, SABiosciences - Qiagen, Germany) was performed as per the manufacturer’s instruction. Eighty-four SYBR Green-optimized primers, related to EMT included in the array, were assessed in a 96-well format. RNeasy mini kit (Qiagen, Germany) was used to purify RNA isolated from stable HCT116 cell lines over-expressing either *Nostrin* cDNA or empty vector. This was followed by concentration and quality checking of the RNA using a Nano Drop 2000 Spectrophotometer (Thermo Fisher Scientific, USA) and fractionation on formaldehyde gel. Genomic DNA elimination was followed by cDNA synthesis using an RT^2^ first strand kit (Qiagen, Germany). The real-time array was then performed using RT^2^ SYBR Green q-PCR Master Mix (Qiagen, Germany). House-keeping genes showing no change in both the groups were used for normalization purpose. Online software provided by SABiosciences was used to calculate the fold change in gene expression.

### Cell invasion assay

A two chambered cell invasion assay kit (ECM550, Chemicon, Merck Millipore, USA) was used to analyze influence of NOSTRIN on cellular invasive activities of colon cancer cells ex vivo. CRC cells as per experimental requirement were treated with 10 μg/ml mitomycin C (Sigma, USA) for 2 h to inhibit cell proliferation and washed extensively. The cells were then seeded onto ECMatrix™ containing trans-well inserts (3 × 10^4^ cells/insert) in serum-free medium. 10% FBS-containing medium kept in the lower chamber served as chemo attractant for the cell migration. Cell migration was allowed for 24 h at 37 °C in 5% CO_2_ followed by removal of non-invaded cells from the interior of the inserts using cotton-tipped swabs. The staining and quantification of the invaded cells were done as described previously [9]. Invaded cells were counted in at least five microscopic fields for each of the three biological replicates. Data represented as mean ± SEM.

### Scratch wound assay

Scratch wound assay in CRC cells was performed as described previously [9]. CRC cells as per experimental requirement were treated with 10 μg/ml mitomycin C (Sigma, USA) for 2 h to inhibit cell proliferation and washed extensively and cultured to form a confluent monolayer, which was wounded by scraping with disposable cell combs (cell comb scratch assay kit, Merck Millipore, USA) to create a “cell-free” area of comparable width. Any detached cell in the wound area was removed by DPBS washing and was cultured for 24 h at 37 °C in 5% CO_2_. Phase contrast images of wounds in multiple fields were taken using Leica microscope (Leica Microsystems, USA), at time t = 0 h, immediately after scratching and also 24 h post scratching. The wound-width were measured by Leica software (LAS X) and the percentage wound healing was calculated using the formula [{(L_0_ – L_24_)/L_0_}× 100] where L_0_ represents the length of the wound at time t = 0 h and L_24_ is the length of the wound at time t = 24 h.

### Soft agar colony formation assay

CRC cells as per experimental requirement were suspended in Dulbecco’s minimal media (DMEM) with 0.4% agar (Cytoselect 96-Well Cell Transformation Assay, Cell Biolabs, USA) and 1 × 10^4^ cells/well were seeded into 96-well culture plates containing 0.6% base agar layer. The cell agar layer was allowed to solidify on the base agar layer and was overlaid with 100 μl of culture medium and incubated at 37 °C in 5% CO_2_ for 7 days. Media was changed every 72 h of the incubation period. After 7 days of incubation colonies were visualized using a Leica microscope (Leica Microsystems) after staining them with 0.05% crystal violet for 15 min. For fluorimetric quantification, unstained colonies were solubilised completely using lysis buffer provided in the kit followed by staining with CyQuant GR dye (1:400). Fluorescence was recorded on a microplate reader (Perkin Elmer, USA) at an excitation wavelength of 485 and emission at 520 nm filter set.

### Colonosphere formation assay

For colonosphere formation stable HCT116 cell lines over-expressing either p3xFLAG-CMV™-10 or p3xFLAG-CMV™-10-NOSTRIN cDNA were trypsinised using 0.25% trypsin, resuspended in serum-free sphere forming medium DMEM/Nutrient Mix F-12HAM(1:1) (Sigma-Aldrich, USA), supplemented with 1X B27 (Invitrogen, USA), 20 ng/ml hEGF (Sigma-Aldrich, USA), 10 ng/ml bFGF (Sigma-Aldrich, USA), 5 μg/ml insulin (Sigma-Aldrich, USA) and 1% Penicillin-Streptomycin (Invitrogen, USA). Cells were cultured in 96 well non-treated cell culture grade plates (Corning, USA) at a density of 200 cells/well for 8 days. Media was changed on every 3.5 days. Colonospheres were harvested on day 8 and evaluated for their number using light microscope (Leica microsystems). The number of colonosphere with diameter greater than 50 μm formed using control and NOSTRIN-overexpressed cells were counted in three replicate wells and the experiment was repeated using three biological replicates.

### BrdU cell proliferation assay

A BrdU cell proliferation kit from Cell Signalling Technology was used to evaluate effect of NOSTRIN on HCT116 cell proliferation as described previously [22]. Stable HCT116 cells over-expressing either the control vector or the *Nostrin* cDNA were seeded (1 X10^4^ cells/well) in triplicates in 96-well plates and cultured for 24 h in the presence of BrdU solution. Cells were then incubated with a fixing/denaturing solution after removing the media, at room temperature for 30 min, followed by incubation of the cells with detection antibody for 1 h. The cells were washed and incubated with HRP-conjugated secondary antibody solution for 30 min. Following washing cells were incubated with tetramethylbenzidine (TMB) substrate, and finally the reaction was stopped after 15 min with a STOP solution. Colorimetric quantification was done by measuring the absorbance at 450 nm wavelength using a multimode plate reader (PerkinElmer, USA).

### Protein isolation, western blotting and immunoprecipitation

Stable HCT116 cell lines over-expressing either *Nostrin* cDNA or empty vector backbone were lysed in radioimmune precipitation buffer supplemented with protease inhibitor mixture (Sigma-Aldrich, USA). Protein concentration for each sample was estimated by using the Bio-Rad Protein Assay reagent (Bio-Rad, USA).

Sixty to 100 μg of total proteins were fractionated by 10–12% SDS-PAGE (Bio-Rad, USA) under reducing condition and were then transferred to PVDF membranes (Millipore, USA). Following blocking and incubation with primary and secondary antibody solution using standard protocol an ECL reagent, Luminata Forte (Millipore, USA) was used for chemiluminescence signal detection using Biospectrum 810 imaging system (UVP, LLC, Upland, CA). Densitometric analysis was done by NIH ImageJ software. Three biological replicates were used for each experiment.

For immunoprecipitation, cell lysates were incubated with either control isotype-matched IgG or anti-NOSTRIN antibody overnight at 4 °C to allow formation of antigen-antibody complex [22]. The antigen-antibody complex was incubated with Pure Proteome protein-A/G mix-magnetic beads (Millipore, USA) for 2 h at room temperature. Elution under denaturing conditions using SDS-PAGE loading buffer was done before gel loading as per manufacturer’s protocol.

### Antibodies

Anti-NOSTRIN (Cat no. ab116374) used at 1:100 dilution and anti-Versican/VCAN (Cat no. ab19345) antibody used at dilution 1:1000 was purchased from abcam, USA. Anti-SNAI2/SLUG (Cat no. 9585), anti-SMAD2 (Cat no. 5339), anti-STAT3 (Cat no. 4904), anti-Caldesmon-1/CALD1 (Cat no. 12503), anti-Jagged1/JAG1 (Cat no. 2620), anti-Moesin/MSN (Cat no. 3150), anti-Vimentin/VIM (Cat no. 5741), anti-Integrinα5/ITGα5 (Cat no. 4705), anti-E-cadherin/CDH1 (Cat no. 3195), anti-Integrin-linked protein kinase1/ILK1 (Cat no. 3856), anti-CD44 (Cat no. 5640), anti-EpCAM (Cat no. 36746), anti-CD133 (Cat no. 86781) and anti-GAPDH (Cat no. 2118) antibodies were purchased from Cell Signaling Technology, USA and was used at 1:1000 dilution. Those procured from Santa Cruz Biotechnology, USA were used at 1:250 dilution, which included anti-Collagen type III Alpha 1 chain/COL3A1 (Cat no. sc-514601), anti-Regulator of G-protein signalling 2/RGS2 (Cat no. sc-100761) and anti-Occludin/OCLN (Cat no. sc-133256). anti-Desmoplakin/DSP (Cat no. A303-355A) and anti-Junctional adhesion molecule A/F11R (Cat no. A302-891A) antibodies were from Bethyl lab, USA. HRP-conjugated goat anti-rabbit and goat anti-mouse antibodies purchased from Bethyl lab, USA were used at 1:10000 dilutions.

### Colon cancer stage-specific cDNA array using clinical samples

Clinical samples across all stages of the disease were acquired from Origene (USA) as Tissue scan colon cancer cDNA array V (Cat No. HCRT105). Colon cancer cDNA array included 48 samples covering 6 normal, 3 stage I, 14 stage IIA, 2 stage IIB, 8 stage IIIB, 8 stage IIIC and 7 stage IV whose clinical and pathological features are available at https://www.origene.com/catalog/tissues/tissuescan/hcrt105/tissue scan-colon cancer-cdna array-v. Quantitative real-time PCR was performed to measure the expression of *Nostrin* in these samples. β-actin was used as an endogenous control and primers used for β-actin were Fwd: 5′-CAGCCATGTACGTTGCTATCCAGG-3′ and Rev.: 5′-AGGTCCAGACGCAGGATGGCATG-3′. Three individual array plates were used for assessing transcript levels of *Nostrin*. The gene expression of *Nostrin* among the different stages of the disease and the normal sample is represented as 2 ^-(ΔCt)^ where ΔCt = Ct_NOSTRIN_ - Ct_β-actin_ for each well.

### Statistical analysis

All data were analyzed by unpaired 2-tailed Student’s t-tests to compare between two independent means, using at least three independent biological replicates. Statistical evaluations were done using Graph pad prism (Version 6.0) software. For all experiments, value of *p* < 0.05 was considered to be statistically significant.

## Results

### Colon cancer cell aggressiveness is inversely related to NOSTRIN expression

**Fig. 1:**
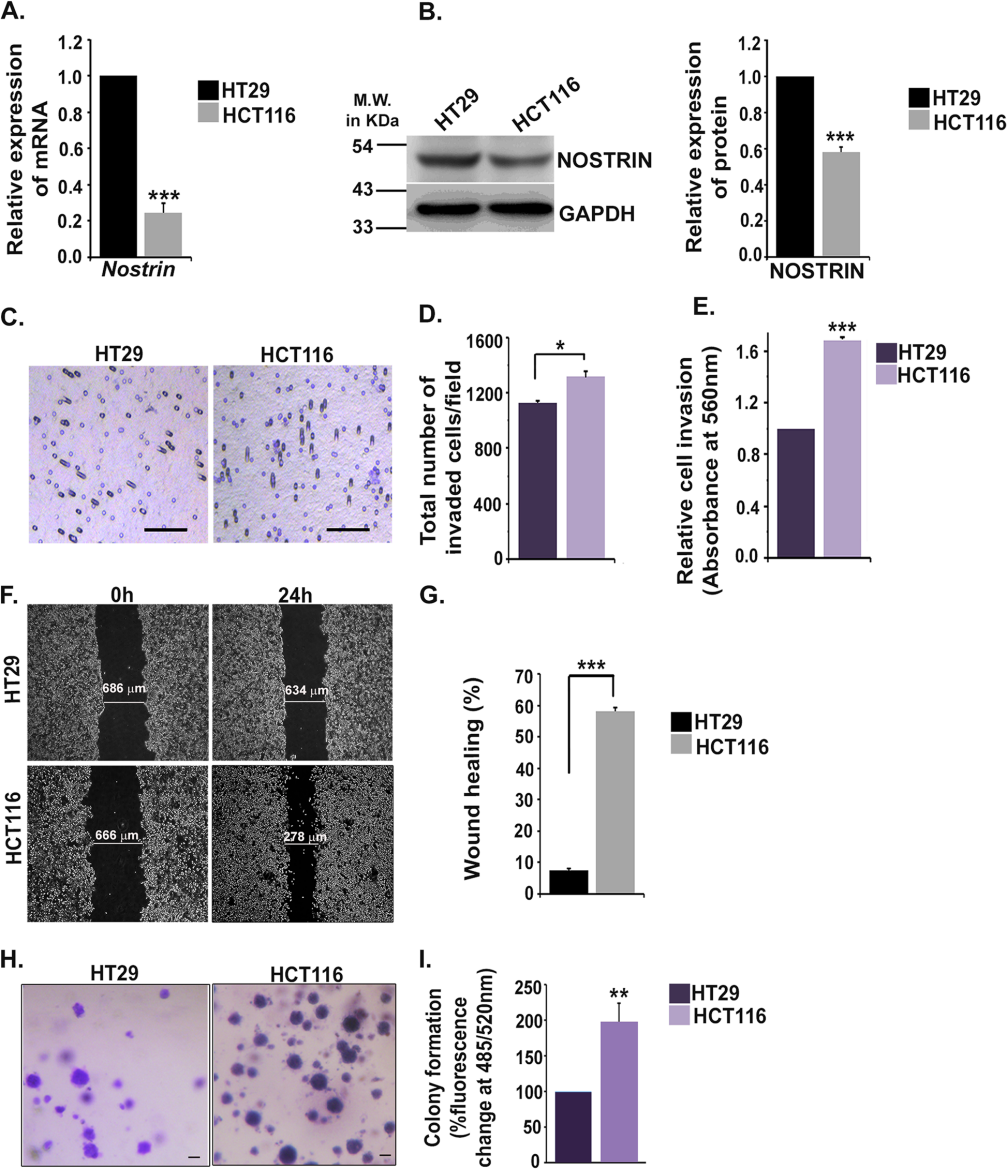
NOSTRIN expression is inversely related to aggressiveness of colon cancer cells. **A** Quantitative real time PCR analysis of *Nostrin* transcript in colon cancer cell lines HT29, HCT116. **B** Western blot analysis of NOSTRIN protein levels in colon cancer cell lines HT29, HCT116. The bar graph adjacent to the blot represents quantification of NOSTRIN protein levels normalized to endogenous control GAPDH using NIH ImageJ software. Full length blots are shown in Fig. S3. **C** Photomicrograph of invaded HT29 and HCT116 cells from cell invasion assay. Scale bar: 100 μm, Magnification: 100X. **D** Invaded cell count per microscopic field. **E.** Colorimetric quantification of the invaded cells at the lower surface of the membrane (*n* = 3). **F** Representative photo-micrographic images depicting wound closure ability of HT29 and HCT116 cell lines. Magnification used, 50X. **G** Quantification of percentage wound closure from a minimum of 5 measurements in each experiment using three different biological replicates. **H** Photomicrographs of colonies formed in soft agar by HT29 and HCT 116 cells. Scale bar: 100 μm, Magnification: 50X. **I** Fluorometric quantification of relative colony formation ability in soft agar of HT29 and HCT116 cells. Data is representative of three independent biological replicates. Error bars represent standard error of mean from three biological replicates. *p < 0.05, ** *p* < 0.01, ***p < 0.001

Anti-angiogenic potentials of NOSTRIN in endothelial cells [9], its high abundance in colon and its role in negatively regulating pancreatic ductal cancer aggressiveness [15] prompted our investigation on association of NOSTRIN expression with colon cancer aggressiveness. Different CRC cell lines like COLO 205, HT29, HCT116 and SW480, which serve as effective cellular model systems [23] were screened for basal NOSTRIN expression levels. COLO 205 and HT29 cells have been categorised as colon like cell types, that expressed higher levels of gastro-intestinal marker genes, along with the miRNA known to repress EMT program [21]. HCT116 and SW480 cells however belonged to an undifferentiated category, characterized by upregulation of EMT and TGFβ induced genes, known to increase tumor initiating potential of CRC cells [21]. NOSTRIN expression was higher in differentiated and colon like cell lines COLO 205 and HT29 compared to the undifferentiated HCT116 and SW480 cells (Fig. S1). Based on the miRNA, mRNA and protein expression studies HCT116 cell line was previously reported to be more aggressive and undifferentiated compared to HT29 cell line [21, 24]. HCT116 cells with reduced NOSTRIN expression and HT29 with maximum (> 2 fold) NOSTRIN expression were chosen for further studies. Expression of *Nostrin* transcripts were approximately 7-fold lower in the more aggressive colon cancer cell line HCT116 compared to the less aggressive HT29 cells (Fig. 1A). Reduced NOSTRIN protein expression in HCT116 cells compared to HT29 cells corroborated the transcript levels (Fig. 1B). Biological functional assays were performed to further ascertain degree of aggression in HCT116 and HT29 cells.

Invasive capability of cells towards chemo-attractant was assessed by cell invasion assay. Both the cell lines were plated separately on trans-well inserts pre-coated with ECM proteins, collagen & elastin and their ability to invade the matrix and attach to the bottom of the insert membrane was scored and quantified. Total number of invaded cells was less using HT29 cell line compared to HCT116 as evident from the representative photographic image (Fig. 1C). There was significant (*p* < 0.05) decrease of approximately 12% in HT29 cells invasion as compared to HCT116 cells (Fig. 1D). Data is representative of average cell count per field analysed out of total 5 fields calculated from each well using three biological replicates. Colorimetric quantification of the stained invaded cells also showed 50% reduction in absorbance in case of HT29 compared to the HCT116 cells (Fig. 1E).

Migration potential of both the cancer cell lines was evaluated using wound healing assay. Photo micrographic images depicting wound at the time of scratching (0 h) and 24 h post-wounding in cells are shown in Fig. 1F. HCT116 cells showed approximately 58% wound healing following 24 h of scratch wounding compared to only 7.5% healing of HT29 cells (Fig. 1G).

Both the cell lines were further assessed for their anchorage independent growth potentials by soft agar colony formation assay. HCT116 cells were found to form more colonies (Fig. 1H) than HT29. Fluorimetric quantification of the stained soft agar colonies showed around 97% increase in fluorescence in HCT116 cells compared to HT29 (Fig. 1I).

### Functional transcriptome analysis reveals the importance of NOSTRIN in regulating Epithelial-Mesenchymal Transition (EMT) associated genes in cancer cells

**Fig. 2:**
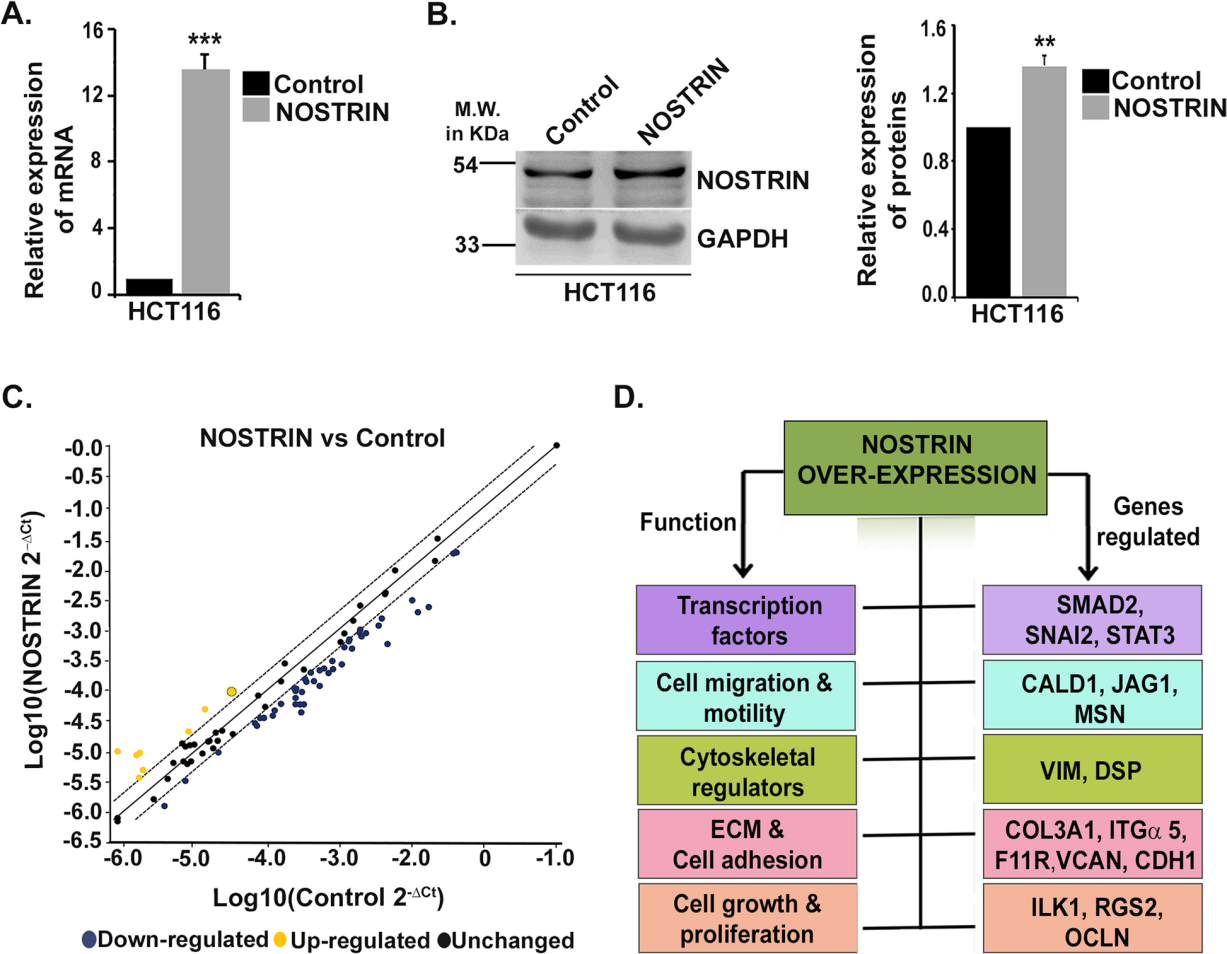
Real time PCR array analysis for profiling Epithelial-Mesenchymal transition (EMT) signature genes in NOSTRIN over-expressed colon cancer cell line. **A** Quantitative real time PCR analysis of *Nostrin* using RNA from empty vector transfected (control) and *Nostrin* cDNA transfected HCT116 cells. GAPDH was used as an endogenous control for normalization. **B** Western blot analysis of NOSTRIN using cell lysates from empty vector transfected (control) and *Nostrin* cDNA transfected HCT116 cells. GAPDH was used as an endogenous control. Full length blots are shown in Fig. S3. Quantification of the protein level from the western blot was done using NIH ImageJ software and is represented using bar graphs adjacent to the blot; Error bars represent standard error of mean from three biological replicates. **p < 0.01, ***p < 0.001. **C** Scatter plot of real time PCR array using control and *Nostrin* overexpressing HCT116 cells showing differential EMT gene expression pattern. Genes with similar expression patterns are represented by black dots that are close to the line of regression while yellow and blue dots are for the up-regulated and down-regulated genes, respectively. **D** Functional annotation of 16 genes that are differentially regulated in all three experiments upon over-expression of NOSTRIN in HCT116 cells

Our further investigations focused on delineating the function of NOSTRIN in affecting colon cancer by over-expressing it in the aggressive HCT116 cell line. HCT116 cells stably over-expressing human *Nostrin* cDNA or the corresponding control with empty vector backbone was established. NOSTRIN over-expression in the HCT116 stable lines was confirmed both in transcript (Fig. 2A) and protein levels (Fig. 2B) which showed significant increase in NOSTRIN expression in the over-expressed group as compared to the control vector expressing ones.

One of the signature events in cancer progression is EMT, an important contributor towards increased cancer invasiveness, migration and metastasis [25]. To assess the downstream effectors of NOSTRIN in EMT regulation we performed a real-time PCR based human EMT RT^2^ Profiler Array comparing HCT116 cells over-expressing NOSTRIN versus control vector. Scatter plot showed changes in expression patterns of eighty-four different genes analysed, which are known to promote EMT (Fig. 2C). Out of the total analysed genes 57 transcripts met the recommended cut-off readings (C_t_ ≤ 30) in one of the two groups. Around 33 transcripts showed significant change (Fold change ≥2, *p* < 0.05) between the two groups analysed in the array (Table 2). The array showed that there was an overall down-regulation of the EMT regulators in NOSTRIN over-expressing cells compared to the control group. The few genes showing up-regulation in the scatter plot (Fig. 2C) did not meet the recommended cut-off in either the C_t_ values or the fold regulation and hence was not considered for further analysis. Only significant up-regulation in the epithelial marker E-cadherin or CDH1 was observed. Based on the functional annotation, differentially expressed transcripts were categorised into five major groups that included a) transcription factors, molecules related to b) cell migration and motility, c) cytoskeletal regulators, d) extracellular Matrix (ECM) & cell adhesion and e) cell growth and proliferation as summarised in Fig. 2D.

**Table 2:** NOSTRIN-induced alteration of transcripts associated with epithelial mesenchymal transition in HCT116 cells

Sl No.	Gene symbol	Accession No.	Mean Ct Control	Mean Ct NOSTRIN	Fold change	Gene description
1	COL3A1	NM_000090	22.60	25.36	-8.2861	Collagen, type III, alpha 1
2	F11R	NM_016946	26.56	29.08	-7.0037	F11 receptor
3	CAV2	NM_001233	26.46	28.64	-5.5639	Caveolin 2
4	FGFBP1	NM_005130	26.63	28.64	-4.9563	Fibroblast growth factor binding protein 1
5	FN1	NM_002026	26.83	28.63	-4.286	Fibronectin 1
6	PTK2	NM_005607	24.69	26.46	-4.1783	PTK2 protein tyrosine kinase 2
7	MSN	NM_002444	25.31	26.93	-3.7788	Moesin
8	CTNNB1	NM_001904	25.11	26.73	-3.7641	Catenin (cadherin-associated protein), beta 1
9	VIM	NM_003380	26.83	28.29	-3.3633	Vimentin
10	TGFB1	NM_000660	26.54	27.96	-3.2979	Transforming growth factor, beta 1
11	TIMP1	NM_003254	25.99	27.38	-3.2095	TIMP metallopeptidase inhibitor 1
12	BMP1	NM_006129	27.88	29.26	-3.1974	Bone morphogenetic protein 1
13	SMAD2	NM_005901	24.23	25.60	-3.1776	SMAD family member 2
14	RGS2	NM_002923	23.02	24.35	-3.0905	Regulator of G-protein signaling 2
15	SNAI2	NM_003068	29.77	27.95	-2.8814	Snail homolog 2 (Drosophila)
16	BMP7	NM_001719	28.59	29.81	-2.846	Bone morphogenetic protein 7
17	ILK1	NM_004517	25.50	26.69	-2.7951	Integrin-linked kinase
18	COL5A2	NM_000393	27.79	28.97	-2.778	Collagen, type V, alpha 2
19	CALD1	NM_004342	25.13	26.28	-2.7295	Caldesmon 1
20	OCLN	NM_002538	23.79	24.94	-2.7118	Occludin
21	ERBB3	NM_001982	27.5	28.61	-2.6688	V-erb-b2 erythroblastic leukemia viral oncogene homolog 3 (avian)
22	AHNAK	NM_024060	28.28	29.38	-2.6367	AHNAK nucleoprotein
23	MAP1B	NM_005909	26.82	27.91	-2.61	Microtubule-associated protein 1B
24	ITGAV	NM_002210	25.71	26.78	-2.5859	Integrin, alpha V (vitronectin receptor, alpha polypeptide, antigen CD51)
25	JAG1	NM_000214	26.34	27.41	-2.567	Jagged 1
26	DSC2	NM_004949	26.8	27.78	-2.4298	Desmocollin 2
27	KRT19	NM_002276	19.42	20.38	-2.3906	Keratin 19
28	STAT3	NM_003150	24.57	25.53	-2.3831	Signal transducer and activator of transcription 3 (acute-phase response factor)
29	NUDT13	NM_015901	28.44	29.38	-2.3525	Nudix (nucleoside diphosphate linked moiety X)-type motif 13
30	ITGα5	NM_002205	26.88	27.768	-2.2717	Integrin, alpha 5 (fibronectin receptor, alpha polypeptide)
31	DSP	NM_004415	23.9	24.74	-2.2066	Desmoplakin
32	VCAN	NM_004385	24.34	25.10	-2.0792	Versican
33	CDH1	NM_004360	27.63	25.73	4.5952	Cadherin 1, type 1, E-cadherin (epithelial)

Array data were validated using quantitative real time PCR taking representative markers from each group described above (Fig. S2).

### Ectopic over-expression of NOSTRIN down regulates Epithelial-Mesenchymal transition (EMT) signature proteins in HCT116 cells

**Fig. 3:**
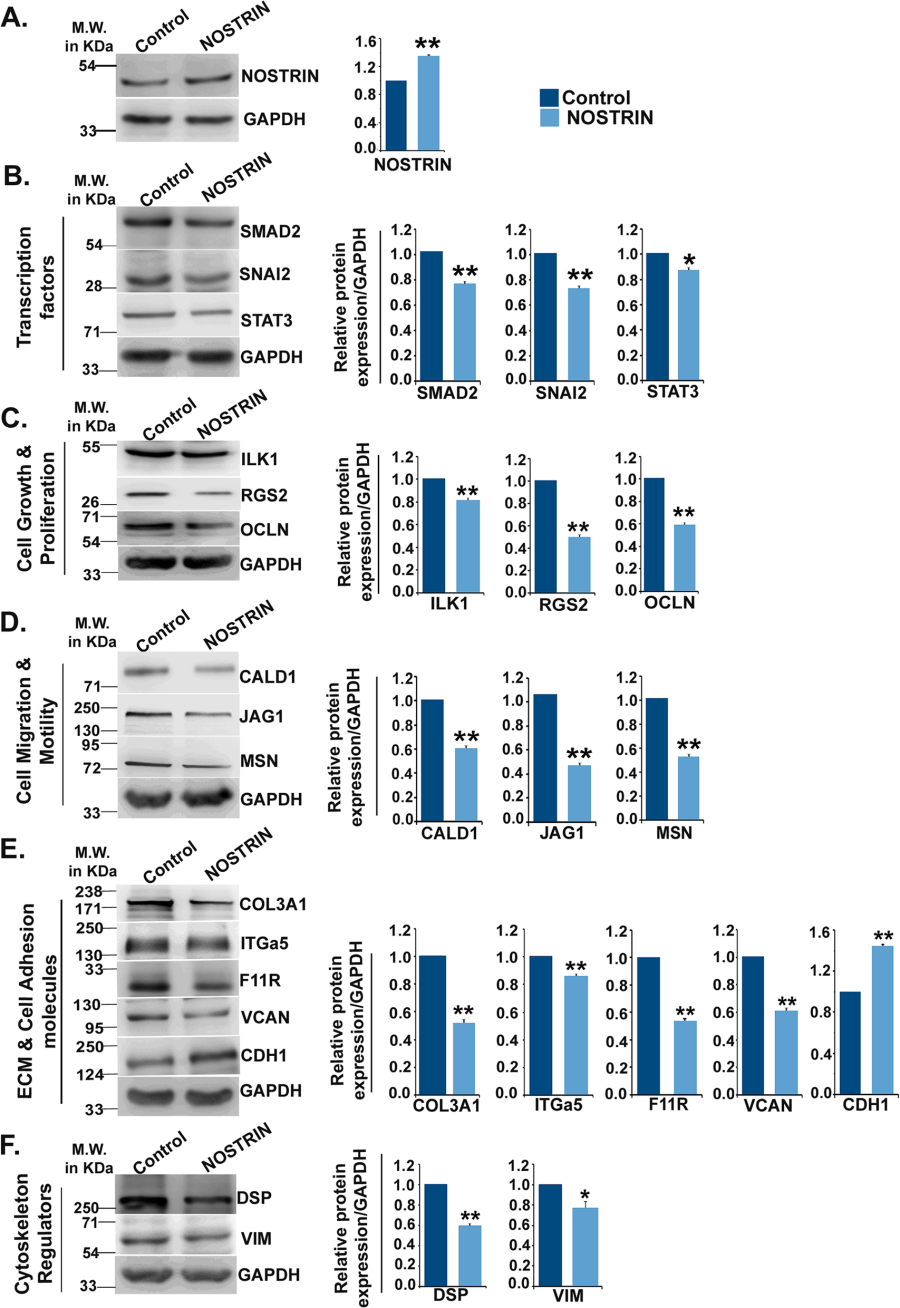
NOSTRIN down-regulates proteins associated with epithelial-mesenchymal transition (EMT) in HCT116 cells. Western-blot analysis of **A** NOSTRIN and **B**-**F** the differentially regulated EMT marker genes in control and NOSTRIN over-expressing HCT116 cells. Western blots are grouped based on functional annotation. **B** Transcription factors: SMAD2, SNAI2 and STAT3. **C** Proteins involved in cell growth and proliferation, ILK1, RGS2 and OCLN **D **Proteins involved in cell migration and motility, JAG1, MSN and CALD1 **E** Proteins associated with extracellular matrix and cell adhesion molecules COL3A1, ITGα5, F11R, Versican (VCAN) and E-cadherin (CDH1). **F** Cytoskeleton regulating proteins, Vimentin (VIM) and desmoplakin (DSP). Quantification of the proteins relative to GAPDH using three biological replicates using NIH ImageJ software has been shown in adjacent bar graphs. Error bars represent standard error of mean from three independent biological replicates. *p < 0.05, **p < 0.01. Full length blots are shown in Fig. S3

Differentially expressed genes identified from the real time PCR array were further validated by evaluating protein levels using immuno-blot assay. Proteins corresponding to sixteen out of the thirty-three transcripts in the array were found to be differentially regulated at the protein level upon NOSTRIN over-expression in all three biological replicates (Fig. 3). NOSTRIN over-expression (Fig. 3A) was found to negatively regulate expression of the transcription factors SMAD2, SLUG (SNAI2) and STAT3 (Fig. 3B), that are known to regulate several EMT related gene expression. There was a simultaneous down-regulation of EMT related proteins associated with cell growth and proliferation that included Integrin linked kinase1 (ILK1), Regulator of G-protein signalling 2 (RGS2) and Occludin (OCLN) (Fig. 3C), along with those that are involved in cell migration and motility comprising of Caldesmon (CALD1), Jagged1 (JAG1) and Moesin (MSN) (Fig. 3D). NOSTRIN over-expression led to down-regulation of ECM and cell adhesion proteins that included Collagen type III alpha1 (COL3A1), Integrin alpha5 (ITGα5), Junctional adhesion molecule A (F11R) and Versican (VCAN) (Fig. 3E). NOSTRIN over-expressed HCT116 cells showed increased expression of the key epithelial marker E-cadherin (CDH1) (Fig. 3E) belonging to the functional group of ECM and Cell adhesion molecule. Potent mesenchymal marker and cytoskeleton regulator Vimentin (VIM) and Desmoplakin (DSP) were down regulated in NOSTRIN over-expressing cells (Fig. 3F). NOSTRIN over-expression thus seemed to suppress overall molecular signatures of EMT in colon cancer cells.

### Oncogenic potential of HCT116 cells is abrogated by NOSTRIN over-expression

**Fig. 4:**
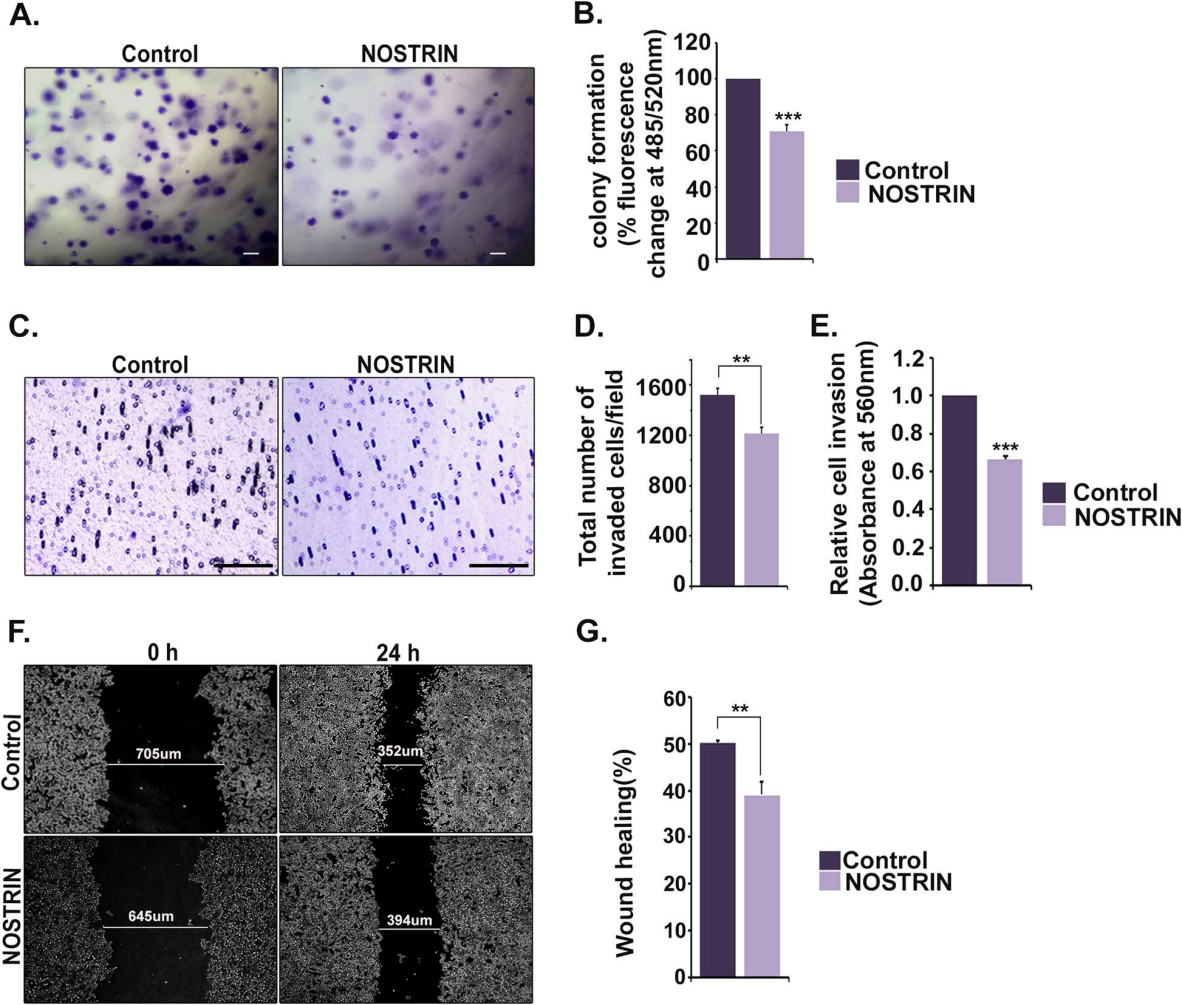
NOSTRIN curtails invasiveness of colon cancer cells. **A** Photomicrographs of colonies formed in soft agar by control and NOSTRIN overexpressing HCT 116 cells. Scale bar: 100 μm, Magnification: 50X. **B** Fluorometric quantification of relative colony formation ability in soft agar of control and NOSTRIN over-expressing HCT116 cells. Data is representative of three independent biological replicates. Error bars represent standard error of mean. **C** Photomicrograph of invaded control and NOSTRIN over-expressing HCT116 cells from cell invasion assay. Scale bar: 100 μm, Magnification: 100X. **D** Invaded cell count per microscopic field. **E** Colorimetric quantification of the invaded cells at the lower surface of the membrane (*n* = 3). **F** Representative photo micrographic images depicting wound closure ability of control and NOSTRIN over-expressing HCT116 cells. Magnification: 50X. **G** Quantification of percentage wound closure from a minimum of 5 measurements in each experiment using three different biological replicates. Error bars represent standard error of mean from three biological replicates. **p < 0.01, ***p < 0.001

Ability of NOSTRIN to down-regulate EMT-associated genes led to investigation on NOSTRIN’s impact in regulating the colon cancer cells to undergo malignant transformation. To assess influence of NOSTRIN on anchorage independent growth soft agar colony formation assay was used. NOSTRIN over-expression in HCT116 cells was found to inhibit colony growth significantly unlike the control group (Fig. 4A). Fluorimetric quantification of the stained soft agar colonies showed around 29% reduction in fluorescence upon NOSTRIN over-expression (Fig. 4B).

NOSTRIN curbed the invasion ability of colon cancer cells towards chemo-attractant. HCT116 cells ectopically over-expressing NOSTRIN or the control vector were plated on Transwell inserts pre-coated with ECM proteins, collagen and elastin. The ability of cells to invade the matrix and attach to the underside of the trans-well membrane was scored as well as quantified. Representative photo-micrographic images of stained cells underside the trans-well are shown in Fig. 4C. There was significant (*p* < 0.01) reduction (19%) in total number of invaded cells upon NOSTRIN over-expression as compared to control (Fig. 4D). Colorimetric quantification of the stained invaded cells also showed ~ 34% reduction in the absorbance upon NOSTRIN over-expression (Fig. 4E).

NOSTRIN’s impact on migration potential of the cancer cells was assessed by scratch wound assay. Representative photo micrographic images depicting wound at the time of scratching (0 h) and 24 h post-wounding in control vector and NOSTRIN over-expressing HCT116 cells are shown in Fig. 4F. Following scratch wounding, cells expressing control vector migrated into the wound and closed nearly 50% of it by 24 h whereas, cells over-expressing NOSTRIN were relatively sluggish and approximately 39% wound was closed by 24 h (Fig. 4G).

### RNA interference of NOSTRIN in HT29 cells enhances its metastatic potential

**Fig. 5:**
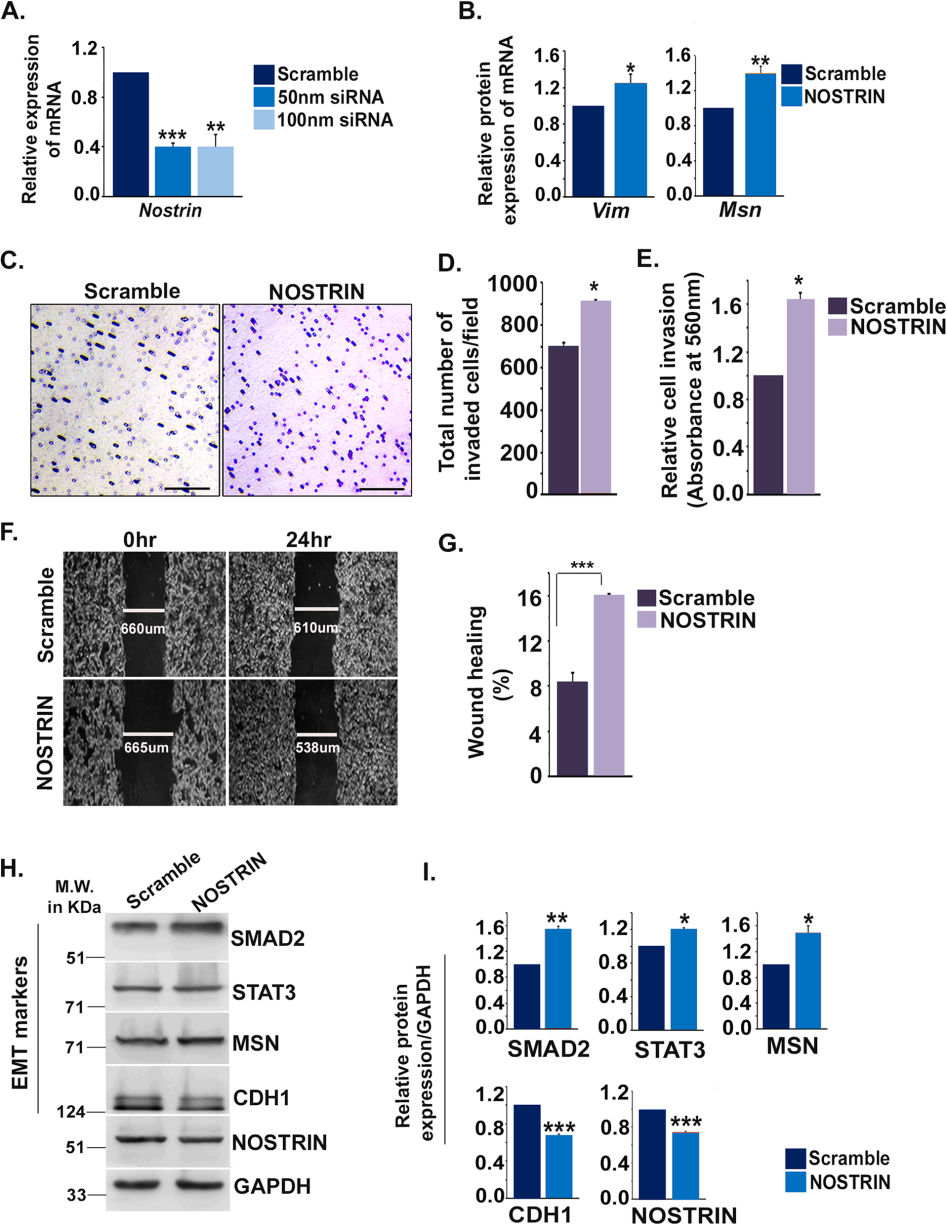
NOSTRIN down-regulation potentiates the metastatic potential of HT29 cells. **A** Quantitative real time PCR analysis of *Nostrin* using RNA from HT29 cells transfected with either scramble or a cocktail of two *Nostrin* siRNAs (50 nM or 100 nM). **B** Quantitative real time PCR analysis of Vimentin *(Vim)* and Moesin (*Msn*) using RNA from HT29 cells transfected with either scramble or 50 nM *Nostrin* siRNA cocktail. GAPDH was used as an endogenous control for normalization. **C** Photo-micrographic images of invaded HT29 cells transfected with either scramble or 50 nM *Nostrin* siRNA cocktail from cell invasion assay. Scale bar: 100 μm, Magnification: 100X. **D** Invaded cell count per microscopic field. **E** Colorimetric quantification of the invaded cells (*n* = 3). **F** Representative photo-micrograph depicting wound closure ability of HT29 cells transfected with either scramble or 50 nM *Nostrin* siRNA cocktail. Magnification used, 50X. **G** Quantification of percentage wound closure 24 h post scratching from a minimum of 5 measurements in each experiment using three different biological replicates. **H** Western-blot analysis of EMT-associated proteins in control and NOSTRIN down-regulated HT29 cells. Full length blots are shown in Fig. S3. **I** Quantification of the proteins relative to GAPDH using three biological replicates using NIH ImageJ software. Error bars represent standard error of mean from three biological replicates. *p < 0.05, **p < 0.01, ***p < 0.001

Two pre-validated Silencer Select siRNAs targeting human NOSTRIN coding regions were used to down regulate *Nostrin* in HT29 cells. A cocktail of 50 nM (25 nM of each siRNAs) was found to be as effective (60%) as 100 nM (50 nM each) in down regulating *Nostrin* (Fig. 5A). The dosage of 50 nM was therefore used in further experiments. HT29 cells possess low levels of migration capacity owing to its low levels of mesenchymal marker expression [26]. Therefore, the expression levels of the mesenchymal marker *Vim* and the cell migration and motility marker *Msn* were evaluated by *Nostrin* RNA interference in HT29 cells. Reduced *Nostrin* expression led to an increase in transcript levels of *Vim (25%)* and *Msn* (40%) in HT29 (Fig. 5B).

The invasive potential of the HT29 cells upon *Nostrin* down-regulation showed remarkable increase as compared to the control group (Fig. 5C). The no. of invaded cells increased by 29.6% in *Nostrin* down-regulated group (Fig. 5D). Colorimetric quantification of the stained invaded cells also showed a significant (*p *< 0.05) increase in the absorbance upon *Nostrin* down-regulation (Fig. 5E). However, migration potential of HT29 cells upon *Nostrin* down-regulation increased marginally (16%) as compared to control group (8%) as demonstrated by scratch wound assay (Fig. 5F and G).

Loss of epithelial marker and gain in mesenchymal markers along with enhanced invasive/migration ability are hallmarks of EMT. To further analyse the influence of reduced *Nostrin* expression on the EMT status of the HT29 cells, expression levels of few EMT signature markers were evaluated. NOSTRIN downregulation led to an increase in the major transcription factors SMAD2 and STAT3 and the potent cell migration and motility marker MSN (Fig. 5H, I). There was a simultaneous significant (*p* < 0.001) decrease in the epithelial marker CDH1 (Fig. 5H, I). Our findings thus clearly confirm role of NOSTRIN down-regulation in enhancing the metastatic potential of HT29 cells.

### NOSTRIN imparts compromised self-renewal ability of colon cancer cells and promotes inhibitory CDK phosphorylation

**Fig. 6:**
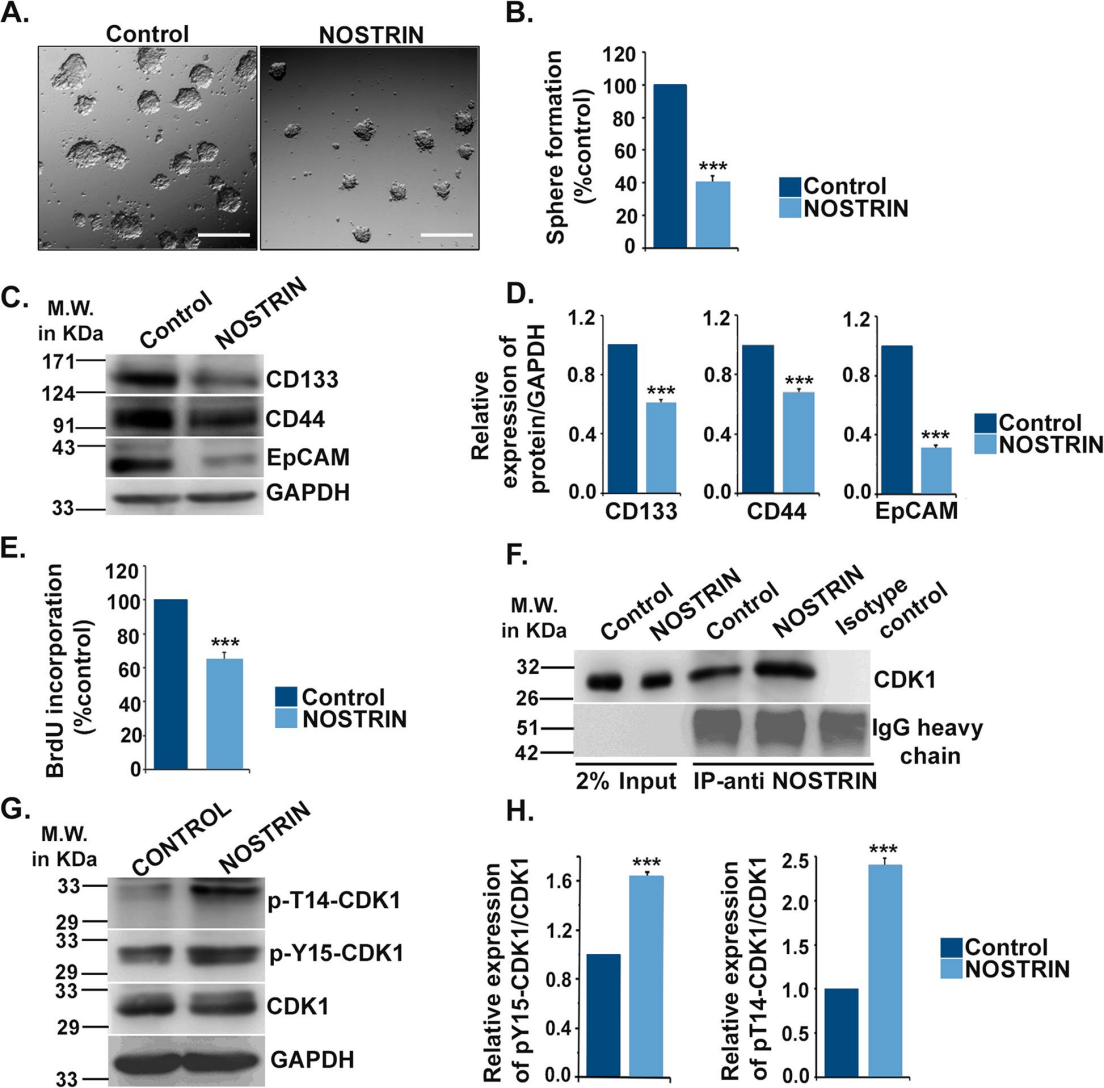
NOSTRIN attenuates self-renewal ability of colon cancer cells and promotes inhibitory CDK phosphorylations. **A** Representative photomicrographs of colonospheres, (magnification 100X, scale bar = 100 μm) formed by control and NOSTRIN over-expressing HCT116 cells. **B** Quantification of number of colonospheres formed by control and NOSTRIN over-expressing HCT 116 cells. **C** Immunoblotting for cancer stem cell markers CD133, CD44 and EpCAM using proteins from control and NOSTRIN over-expressing HCT 116 cells. Full length blots are shown in Fig. S3. **D** Quantification of blots in C using using NIH Image J software. GAPDH was used as endogenous control. **E** BrdU incorporation assay scoring cell proliferation in control and NOSTRIN over-expressing HCT116 cells. **F** Immuno-precipitation (IP) with anti-CDK1 antibody followed by western blotting using anti-NOSTRIN antibody in control and NOSTRIN over-expressing HCT116 cells. Full length blots are shown in Fig. S3. **G** Western blot analysis of p-T14-CDK1, p-Y15-CDK1 and CDK1 using control and NOSTRIN over-expressing HCT 116 cells. GAPDH was used as an endogenous control. Full length blots are shown in Fig. S3. **H** Quantification of the phosphoproteins (T14 & Y15) relative to basal CDK1 levels from blots in G using NIH Image J software. Full length blots are shown in Fig. S3. Error bars represent standard error of mean from three independent biological replicates. ***p < 0.001

During cancer progression, a population of cancer cells possessing self-renewal potentials referred to as cancer stem cells (CSC) exist that contributes to cancer drug resistance and disease recurrence [27]. Previous reports on association of NOSTRIN with Cdk1/cdc2 leading to inhibition of cell cycle progression [12] prompted our investigation on effect of NOSTRIN in regulating stemness of colon cancer cells using colonosphere formation assay. NOSTRIN abrogated colonosphere formation ability of HCT116 cells as shown in representative photo micrographic image (Fig. 6A). Quantification of colonosphere formation showed that there was 60% reduction in sphere formation in NOSTRIN over-expressing HCT116 cells as compared to controls (Fig. 6B).

CSCs are characterized by expression of surface markers that includes CD133, CD44 and EpCAM which are of specific relevance to colorectal cancer [27]. NOSTRIN over-expression was found to down-regulate the potent CSC surface markers CD133, CD44 and EpCAM (Fig. 6C and D) in HCT116 cells compared to the control cells.

Cancer cells are characterized by abnormal cell cycle leading to enhanced cell proliferation and uncontrolled growth. To assess the effect of NOSTRIN on proliferative potential of colon cancer cells, BrdU incorporation assay was performed. Ectopic over-expression of NOSTRIN in HCT116 cells significantly (*p* < 0.001) decreased BrdU incorporation by 44% as compared to control (Fig. 6E).

To elucidate the mechanism by which NOSTRIN might inhibit colon cancer cell proliferation, it was found NOSTRIN might halt cytokinesis owing to its structural similarities with Hof1 [28]. Besides, previous report [12] demonstrated that in differentiating trophoblast cells NOSTRIN interacts with Cdk1, which is a component of mitosis promoting factor [29]. Physical interaction of NOSTRIN with Cdk1 was therefore, tested in colon cancer cells using immunoprecipitation with anti-Cdk1 (Cdc2) antibody followed by western blotting using anti-NOSTRIN antibody. NOSTRIN was found to form immune-complex with Cdk1 and this interaction was augmented in HCT116 cells overexpressing NOSTRIN (Fig. 6F). Phosphorylation on T14 and Y15 residues of Cdk1 arrests mitosis [30]. In line with this, it was observed that phosphorylation of both T14 and Y15 residues of Cdk1 significantly (*p* < 0.001) increased in NOSTRIN over-expressing HCT116 cells compared to the control vector (Fig. 6G and H). Thus, interaction between NOSTRIN and Cdk1 indicated a plausible mechanism behind increased inhibitory effect on Cdk1 and the proliferative potentials of colon cancer cells.

### Colon cancer progression through various stages is associated with reduced NOSTRIN mark

**Fig. 7:**
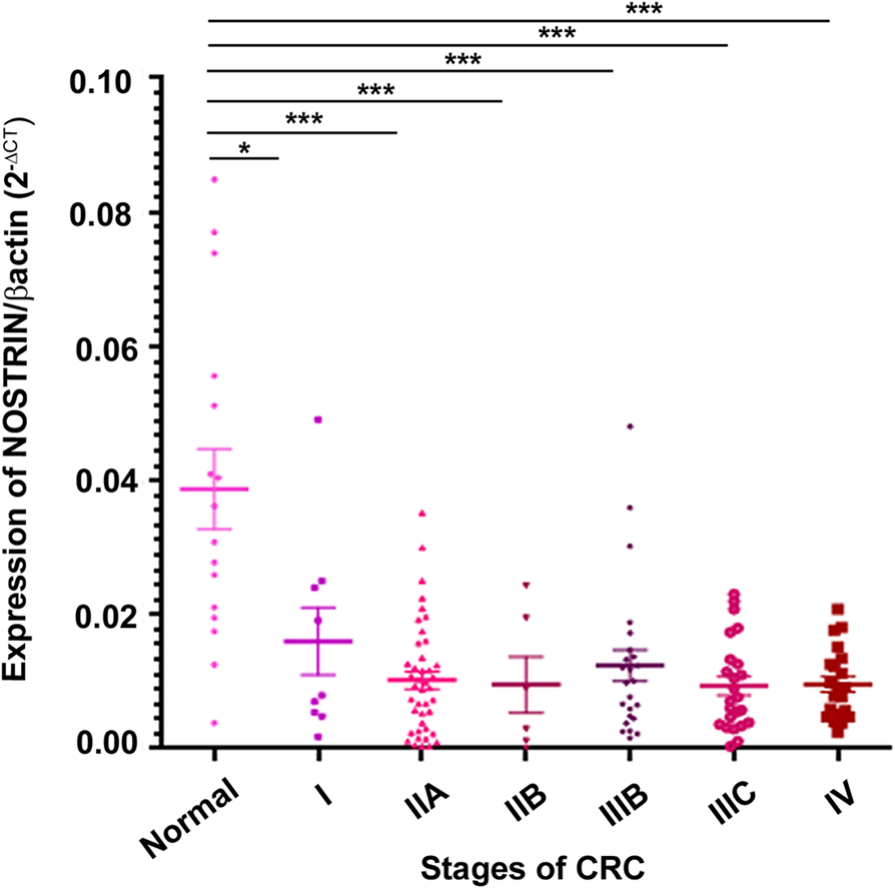
Expression of *Nostrin* transcripts in colon cancer patients at different stages of disease progression. Quantitative real time PCR analysis of *Nostrin* transcripts with 144 clinical samples stratified based on tumour stage using TissueScan™ qPCR array. β-actin was used as an endogenous control. *p < 0.05, ***p < 0.001

To corroborate our *ex vivo* findings with *in vivo* colon cancer progression, we performed a real time PCR based human colon cancer cDNA array (OriGene, USA) containing cDNAs from different stages of colon cancer patients including those from normal colon samples, comparing changes in *Nostrin* transcripts among those patient samples. Onset of the CRC was found to be associated with significantly (*p* < 0.05) reduced *Nostrin* transcripts as compared to control samples (Fig. 7). *Nostrin* transcripts were found to decrease with advancement of the disease stages reaching its minimal level at stage IIB (*p* < 0.001), following which it plateaued over stages IIIB, IIIC and IV. Thus, stages beyond IIB showed similar reduction in *Nostrin* levels as stage IIB compared to normal colon.

**Fig. 8:**
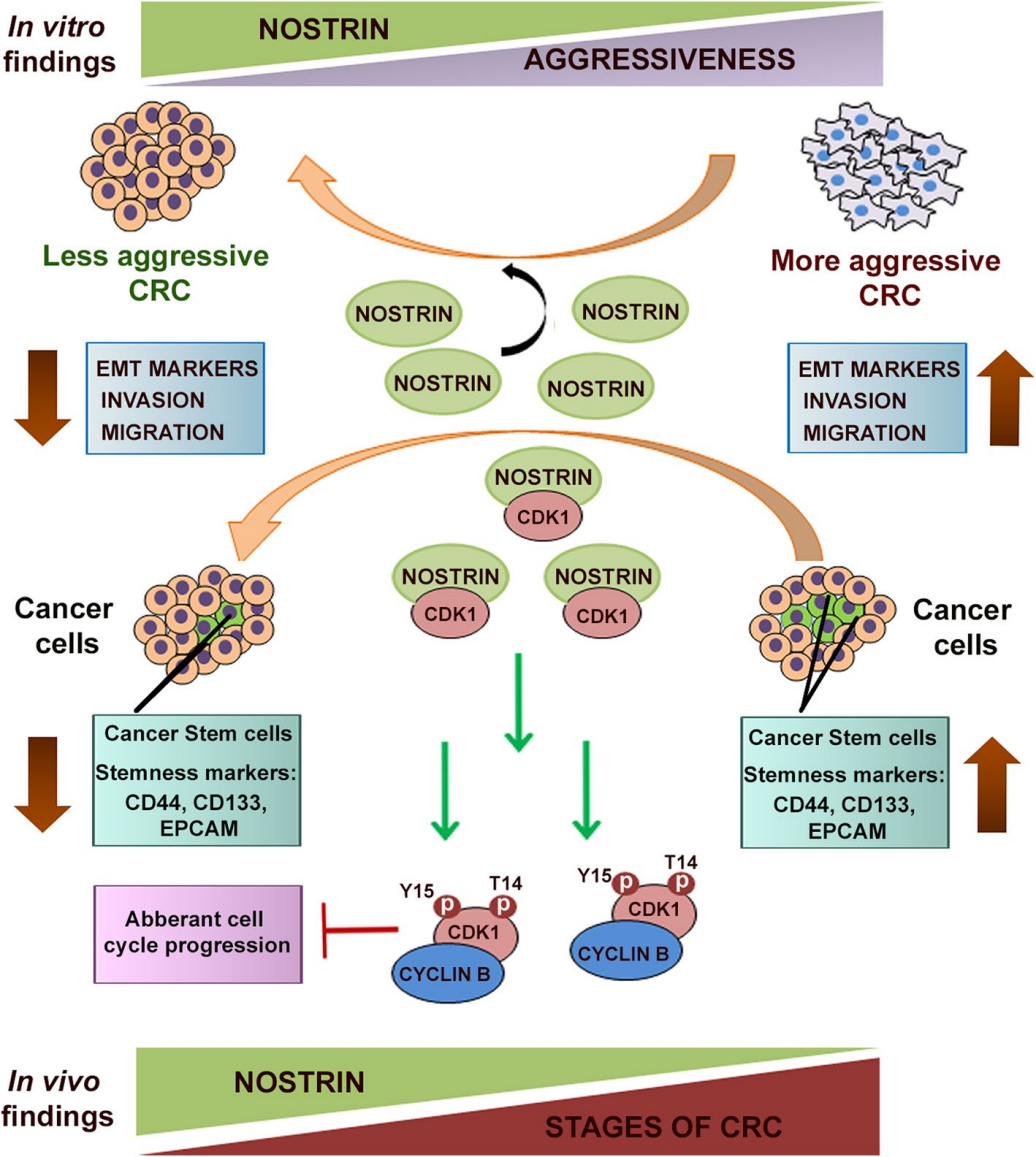
A schematic representation of NOSTRIN mediated regulation of the colon cancer aggressiveness. NOSTRIN expression shows an inverse relation with the colon cancer aggressiveness and hence ectopic over-expression of NOSTRIN leads to reduction in their aggressive phenotype as evident from alterations in the EMT programme, invasive, migratory and stemness properties. Interaction of NOSTRIN with CDK1 leading to increased inhibitory CDK1 phosphorylation stalls aberrant cell cycle proliferation in colon cancer cells and presumably their stemness. Reduced *Nostrin* mark is a hallmark of CRC disease progression

Taken together, our findings demonstrate high NOSTRIN expression can reduce aggressiveness of colorectal cancer (CRC) cells through attenuation in the EMT programme and their metastatic potentials. Increased NOSTRIN expression leads to decreased stemness of cancer cells. Interaction of NOSTRIN with Cdk1 is associated with increased inhibitory Cdk phosphorylation thereby restricting cancer cell proliferation. Onset of CRC as well as disease progression is associated with highly reduced *Nostrin* transcripts. The overall regulatory effect of NOSTRIN on CRC progression is represented schematically in Fig. 8.

## Discussion

Induction of EMT not only plays an important role in cancer progression but also is associated with generation of cancer stem cells [31, 32]. It is evident that a myriad of genes participates and cooperates with each other throughout cancer progression. Although the relevance of decreased NOSTRIN expression with disease progression in pancreatic adenocarcinoma has been well documented [15], role of NOSTRIN in CRC has not been reported despite its abundance in colon. Association of NOSTRIN expression with aggressiveness of colon cancer cell lines, the genetic profile of EMT regulated by NOSTRIN in CRC cells as well as NOSTRIN’s ability in regulating stemness of CRC are yet to be elucidated. In this report, we concisely illustrated the regulatory role of NOSTRIN in CRC progression *ex vivo* using relevant CRC cells lines and also elucidated the correlation of NOSTRIN expression with progression of CRC using normal colon tissue as control.

Reduced expression of NOSTRIN in the aggressive HCT116 cells belonging to the undifferentiated category compared to the colon-like subtype HT29 cells was evident from our data. Interestingly, this *ex vivo* data, clearly recapitulated with NOSTRIN mark in the patient samples using cDNA array data. HCT116 represents a highly aggressive and most commonly used cell line with no ability to differentiate [24] and hence was chosen to delineate effect of NOSTRIN over-expression in combating the disease aggressiveness. Our data clearly put forth the ability of NOSTRIN in repressing the EMT program.

Our data showed that NOSTRIN effectively repressed expression of EMT-inducing transcription factors. SNAI2 was shown to impart 5-FU chemotherapy resistance in CRC patients [33]. Our data on NOSTRIN-induced suppression of SNAI2 and elevation of E-cadherin (CDH1) in CRC cells is supported by SNAI2’s ability to repress transcription of E-cadherin (CDH1) [34]. STAT3, known to positively regulate EMT enhances proliferation or reduces apoptosis in various types of cancer [35–37] thereby promoting cancer progression. Constitutive activation of STAT3 in colon cancer cells leads to cell proliferation and tumor growth [38]. Thus, NOSTRIN-mediated decrease in STAT3 expression could be just another step towards reduced colon cancer aggressiveness. Activation of TGF-β1-mediated SMAD2/3 signalling has been reported to induce EMT thereby promoting carcinogenesis in Oesophageal Squamous Cell Carcinoma patients [39]. Our data on NOSTRIN-mediated down regulation of SMAD2 thereby indicates significance of NOSTRIN in down-regulating EMT in CRC cells.

Regulators of cell growth and proliferation plays a very important role in inducing EMT that includes Integrin linked kinase1 (ILK1), Occludin (OCLN) and Regulator of G-protein signalling 2 (RGS2). ILK1 over-expression has been reported to be responsible for aggressive features and chemo-resistance in human colon cancer [40]. Inhibition of Occludin led to decreased cell proliferation, invasion, migration and induced apoptosis in lung cancer cells [41]. Similarly, RGS2 was shown to be up-regulated in the patients with early onset colorectal cancer [42]. Our data on NOSTRIN-induced down-regulation of ILK1, Occludin and RGS2, therefore, re-confirm NOSTRIN’s role in suppressing CRC progression.

EMT is characterized by significant changes in cytoskeletal organization accompanied by enhanced expression of actin associated proteins that regulate various functions of cell motility. Caldesmon (CALD1) is one such actin linked regulatory protein that is up-regulated following EMT induction [43]. Enhanced expression of CALD1 imparts resistance to chemotherapeutic drug (5-FU) and radiation treatment in colorectal cancer cells [44]. Moesin (MSN) belonging to ERM family of proteins has been shown to be associated with progression of different cancer types including papillary thyroid carcinomas [45], glioblastoma tumors [46], pancreatic cancers [47] and colorectal carcinoma [48]. Elevated JAG1 leads to abnormal up-regulation of its receptor Notch-1 accompanied by inhibition of tumor cell apoptosis, development and metastasis of tumors [49]. In line with this, our data on NOSTRIN-induced down- regulation of CALD1, JAG1 and MSN indicate NOSTRIN’s inhibitory function in CRC.

Extracellular matrix (ECM) proteins play a very important role in regulating EMT program affecting tissue remodelling and cancer tumorigenesis [50]. Collagen type III (COL3A1) has been reported to promote cell proliferation, metastasis and invasion [51, 52] and is increased in colorectal cancer patients [53]. Similarly, Integrin alpha 5 (ITGα5) expression is enhanced with higher CRC progression, metastasis, decreased cell apoptosis [54] and increased EMT [55]. RNA–Seq data of the Cancer Genome Atlas cohort correlated high expression of ITGα5 with poor overall survival in colorectal adenocarcinoma that was further confirmed in an independent study of 355 patients [56]. Junctional adhesion molecule A or F11R being an important junctional adhesion molecule is known to play a vital role in cell migration, invasion and adhesion contributing to carcinogenesis [57] and has been reported to be over-expressed in kidney, lung, and breast tumor tissues [58]. Another important ECM protein, Versican (VCAN) is known to be up-regulated in a wide range of cancers [59] and has been reported as a strong prognostic marker in stages II and III colon cancer [59, 60]. NOSTRIN-mediated down-regulation of these important ECM and cell adhesion molecules as evident from our present study clearly indicates the significance of NOSTRIN in limiting the EMT program in CRC cells.

Mesenchymal marker Vimentin up-regulation is considered to be one of the best indicators of EMT in tumorigenesis [61]. Evidences support role of Vimentin over-expression in stimulating metastasis and invasion in colorectal cancer [62]. Our data showing NOSTRIN-induced suppression of the mesenchymal marker Vimentin further confirms NOSTRIN-mediated suppression of EMT in CRC cells.

Ectopic over-expression of NOSTRIN in HCT116 cells not only restricted the EMT program, but also reduced the oncogenic potentials of the cells. Thus, NOSTRIN over-expression led to decrease in the migration, invasion and the anchorage independent growth potentials of the aggressive HCT116 cells. The protective role of NOSTRIN was further re-affirmed by RNA interference of *Nostrin* in HT29 cells. Functional assays reported here demonstrated that *Nostrin* down-regulation could effectively enhance the aggressiveness of the HT29 cells. NOSTRIN down-regulation caused up-regulation of the mesenchymal markers with decrease in the potent epithelial marker CDH1. Thus, NOSTRIN expression level determines the extent of CRC aggressiveness.

A direct link between EMT and gain of epithelial stem cell property has been reported earlier [32]. EMT and cancer stem cells together contribute to a more aggressive, metastatic tumor progression [63, 64]. Interestingly, our data demonstrated that NOSTRIN over-expression not only suppressed EMT program, but it also led to reduction in colonosphere forming ability and cancer stem cell marker expression in the colon cancer cells. CD44, a cancer stem cell marker has been reported to get over-expressed in colorectal adenomas thereby driving them towards carcinoma [65, 66]. Another well known CSC marker is EpCAM expressed by cancers of epithelial origin and its over-expression is associated with poor survival and advanced disease stage [27]. EpCAM^high^/CD44^+^ population were shown to be more effective in developing tumours in non-obese diabetic/SCID mice [65]. Our data corroborates with these findings demonstrating that NOSTRIN could effectively down-regulate expression of both these potent stem cell markers, CD44 and EpCAM. These observations clearly suggest potential role of NOSTRIN in negatively regulating colon cancer progression by suppressing both the EMT program and the colon cancer stem cell properties.

Our data on NOSTRIN’s ability to decrease the proliferative potential of HCT116 colon cancer is rather intriguing. Structural similarity of NOSTRIN with yeast F-BAR protein HOF1, a potent cytokinesis and mitosis promoting complex inhibitor [27] supports our observation. This is in line with our data on NOSTRIN’s ability to interact with Cdk1 and thereby increase in inhibitory phosphorylation of T14 and Y15 residues of Cdk1. This data signifies destabilising effects of NOSTRIN over-expression on mitosis promoting complex.

Our data on human patient samples established a negative correlation between NOSTRIN expression and advanced disease stages of colon cancer. Patients with early stage colon cancer i.e. stage I & IIA, where according to the surgical stage grouping the cancer is restricted to the walls of the colon and has not spread to nearby lymph nodes or distant organs, showed higher NOSTRIN expression compared to advanced disease stages i.e. stage IIB, IIIB, IIIC (our data), where the tumor has spread to nearby lymph nodes & stage IV where the tumor has also spread to some distant organs along with the lymph nodes. The array data further established that there is remarkable decrease in NOSTRIN expression right at the onset of the disease as it was evident from our results that early stages of the colon cancer showed reduced NOSTRIN expression compared to the normal colonic samples indicating its probable role as a marker of disease aggressiveness.

## Conclusion

This report established a significant role of NOSTRIN in inhibiting a) EMT, b) metastatic potential and c) self-renewal of CRC cells. It can be inferred from our data that remarkable down-regulation of NOSTRIN expression marks the onset of CRC. In addition, CRC disease progression is associated with very low levels of NOSTRIN expression. We predict that NOSTRIN mark may be used as an early marker for colorectal cancer progression and metastasis.

## Supplementary Information

**Additional file 1: Fig. S1.** NOSTRIN protein expression in various CRC cell lines. A. Western-blot analysis of NOSTRIN in different CRC cell lines. B. Quantification of the protein bands from (A) using NIH Image J software. GAPDH was used as endogenous control. Error bars represent standard error of mean from three independent biological replicates. **p < 0.01, ***p < 0.001.

**Additional file 2: Fig. S2.** Influence of NOSTRIN on EMT signature transcripts in HCT116 cells. A. Quantitative real time PCR analysis of *Smad2, Stat3, Vim, Msn*, *Col3a1, Itgα5, F11r and Vcan* using RNA from HCT116 cells transfected with either control vector of *Nostrin* cDNA. GAPDH was used as an endogenous control for normalization. Error bars represent standard error of mean from three independent biological replicates. *p < 0.05, ***p < 0.001.

**Additional file 3: Fig. S3.** Original full-length blots are compiled and shown along with their corresponding cropped versions.

## Data Availability

Data and materials are available on request from the corresponding author.

## Abbreviations

NOSTRIN: Nitric-Oxide Synthase trafficking inducer
CRC: Colorectal cancer
EMT: Epithelial-mesenchymal transition
CD133: Prominin-1
CD44: CD44 molecule (Indian Blood Group)
EpCAM: Epithelial cell adhesion molecule
BrdU: Bromodeoxyuridine
Cdk1: Cyclin-dependent kinase-1
Y15: Tyrosine-15
T14: Threonine-14
cDNA: complimentary DNA
eNOS: Endothelial Nitric Oxide Synthase
NO: Nitric oxide
PDAC: Pancreatic ductal adenocarcinoma
CO_2_: Carbon dioxide
M-MLV: Moloney Murine Leukemia virus
G418: Geneticin
PCR: Polymerase chain reaction
Fwd: Forward
Rev.: Reverse
ECM: Extracellular matrix
FBS: Fetal bovine serum
SEM: Standard error mean
DPBS: Dulbecco’s phosphate buffered saline
TMB: Tetramethylbenzidine
ECL: Enhanced chemiluminiscence
IP: Immunoprecipitation
VCAN: Versican
SNAI2: Snail family transcriptional repressor 2
SMAD2: SMAD family member 2
STAT3: Signal transducer and activator of transcription 3
CALD1: Caldesmon 1
JAG1: Jagged1
MSN: Moesin
VIM: Vimentin
CDH1: Cadherin1
ITGα5: Integrinα5
ILK1: Integrin-linked protein kinase1
COL3A1: Collagen type III Alpha 1 chain
RGS2: Regulator of G-protein signalling
OCLN: Occludin
DSP: Desmoplakin
F11R: Junctional adhesion molecule A
HRP: Horseradish peroxidase
miRNA: Micro RNA
CSC: Cancer stem cell
Cdc2: Cell division cycle protein 2
Hof1: Homologue of fifteen
FU: Fluorouracil
SCID: Severe combined immunodeficiency
