# Supplementary figures and images for: Nitric-Oxide Synthase trafficking inducer (NOSTRIN) is an emerging negative regulator of colon cancer progression
Source: BMC Cancer. 2022 May 31;22:594. doi: 10.1186/s12885-022-09670-6 (PMC9158178; doi:10.1186/s12885-022-09670-6)

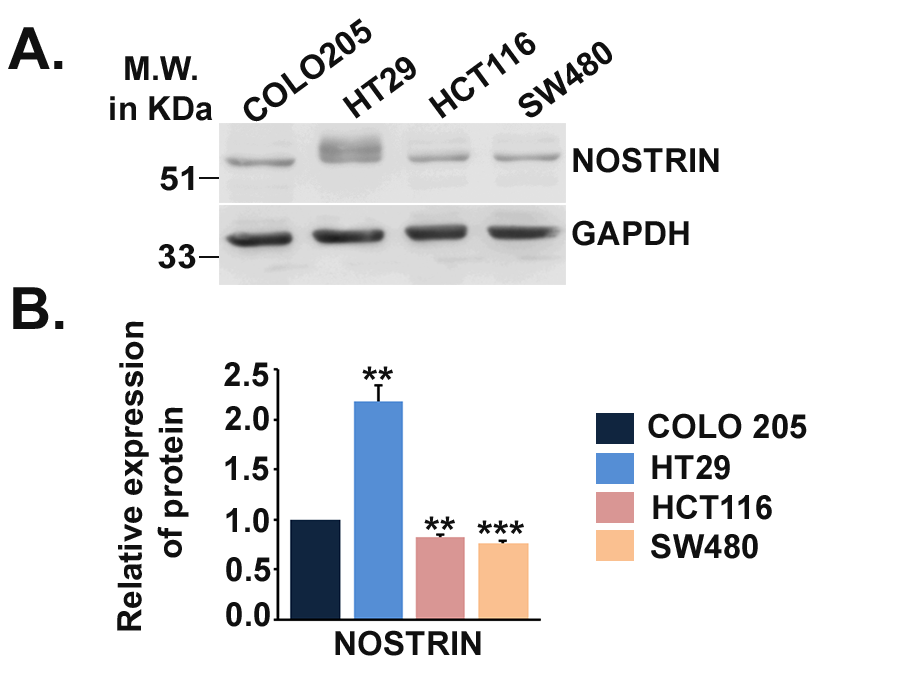

Supplement: Supplementary file 1 — Additional file 1: Fig. S1. NOSTRIN protein expression in various CRC cell lines. A. Western-blot analysis of NOSTRIN in different CRC cell lines. B. Quantification of the protein bands from (A) using NIH Image J software. GAPDH was used as endogenous control. Error bars represent standard error of mean from three independent biological replicates. **p < 0.01, ***p < 0.001. [file 12885_2022_9670_MOESM1_ESM.tif]

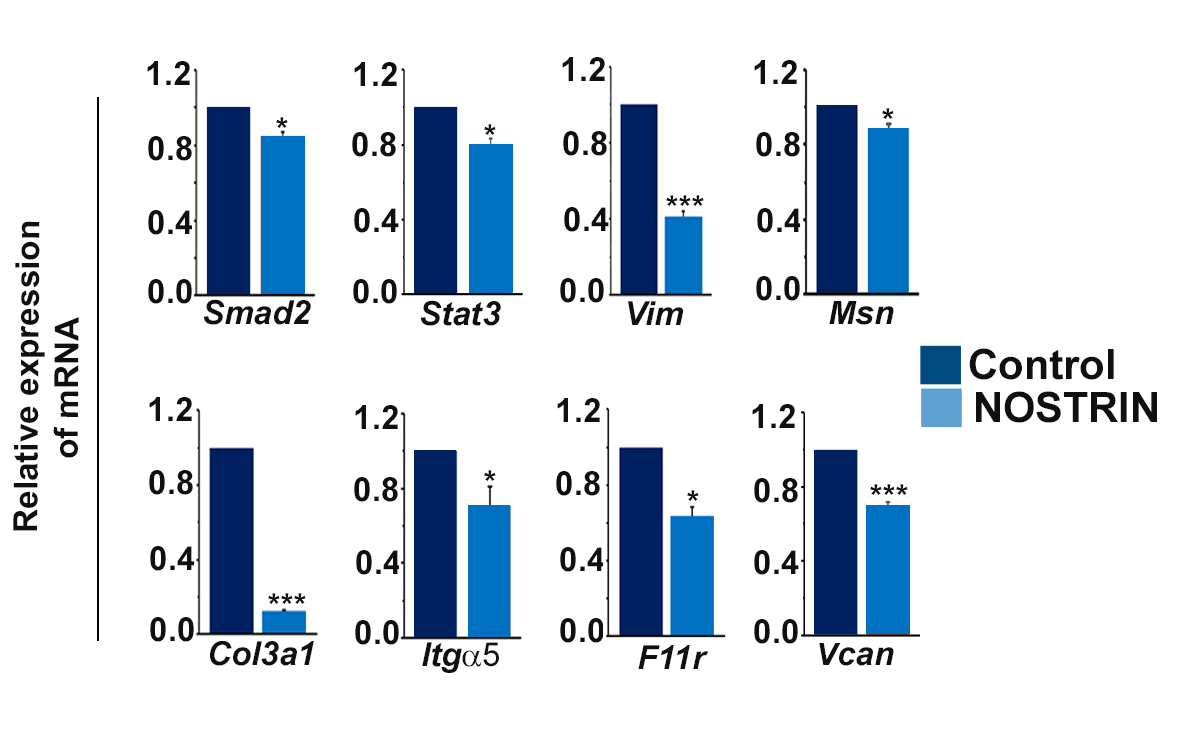

Supplement: Supplementary file 2 — Additional file 2: Fig. S2. Influence of NOSTRIN on EMT signature transcripts in HCT116 cells. A. Quantitative real time PCR analysis of Smad2, Stat3, Vim, Msn, Col3a1, Itgα5, F11r and Vcan using RNA from HCT116 cells transfected with either control vector of Nostrin cDNA. GAPDH was used as an endogenous control for normalization. Error bars represent standard error of mean from three independent biological replicates. *p < 0.05, ***p < 0.001. [file 12885_2022_9670_MOESM2_ESM.tif]
