# Supplementary material for: Nitric-Oxide Synthase trafficking inducer (NOSTRIN) is an emerging negative regulator of colon cancer progression
Source: BMC Cancer. 2022 May 31;22:594. doi: 10.1186/s12885-022-09670-6 (PMC9158178; doi:10.1186/s12885-022-09670-6)

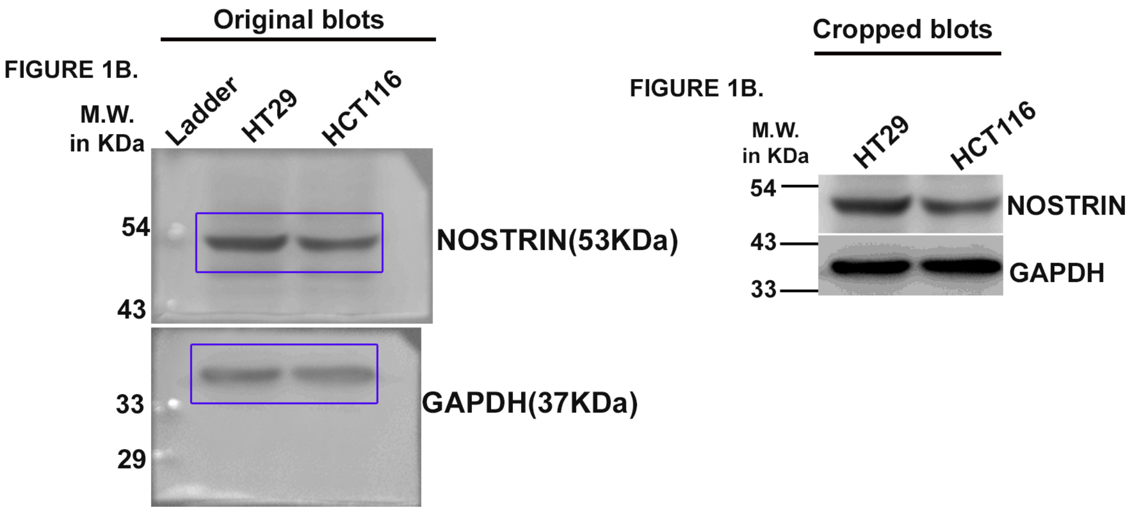
Original blots
FIGURE 1B.
M.W. in KDa
Ladder
HT29
HCT116
54
43
33
29
NOSTRIN(53KDa)
GAPDH(37KDa)
Cropped blots
FIGURE 1B.
M.W. in KDa
HT29
HCT116
54
43
33
NOSTRIN
GAPDH

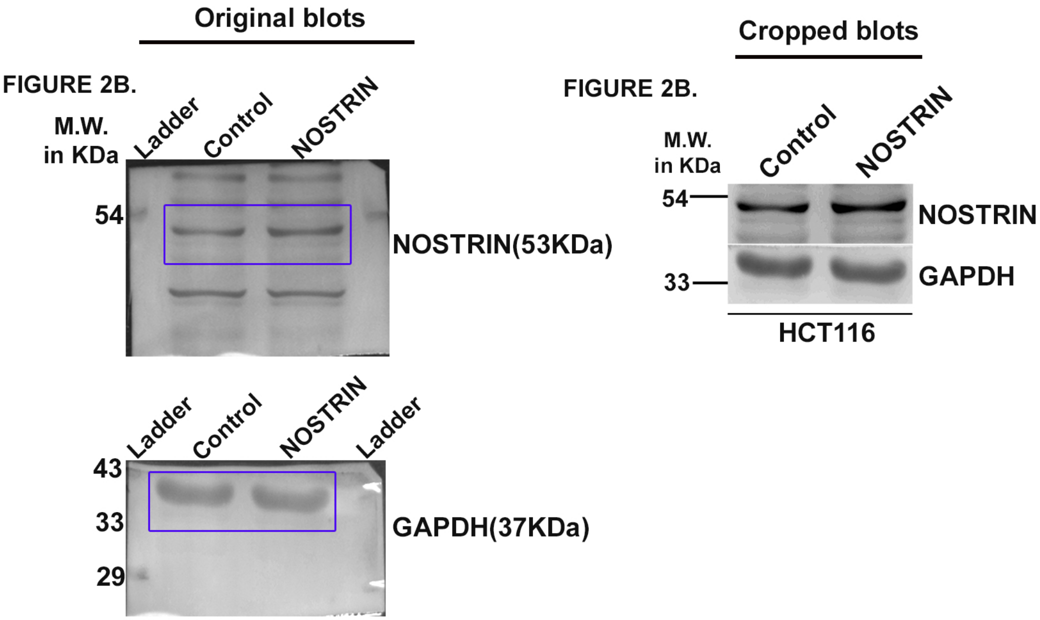
Original blots
FIGURE 2B.
M.W. in KDa
Ladder
Control
NOSTRIN
54
NOSTRIN(53KDa)
Ladder
Control
NOSTRIN
Ladder
43
33
29
GAPDH(37KDa)
Cropped blots
FIGURE 2B.
M.W. in KDa
Control
NOSTRIN
54
33
NOSTRIN
GAPDH
HCT116

Original blots

FIGURE 3A.

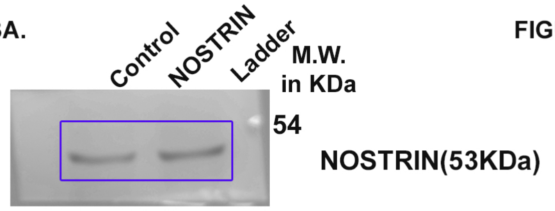

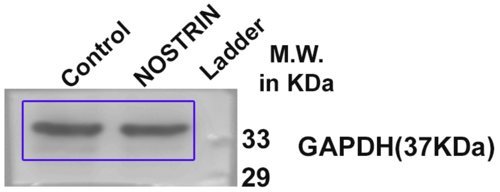

FIGURE 3B.

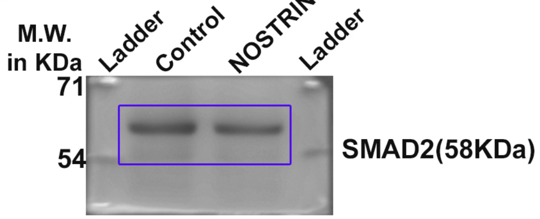

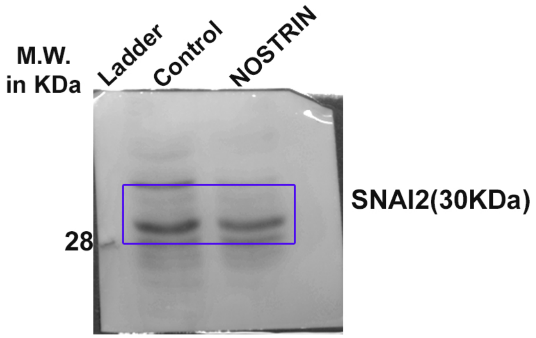

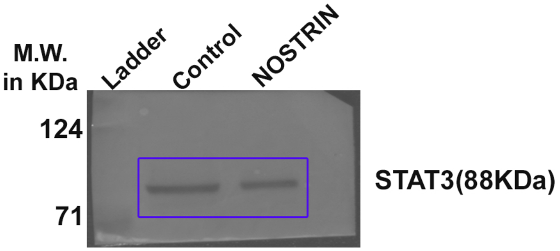

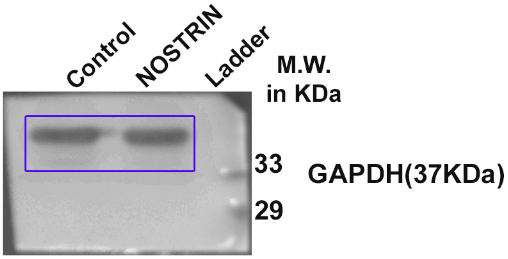

Cropped blots

FIGURE 3A.

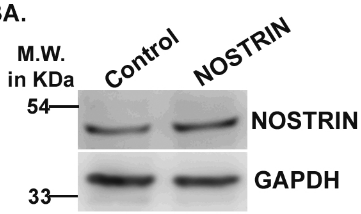

FIGURE 3B.

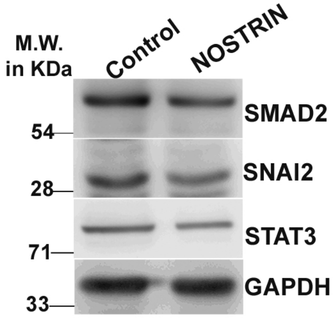

Original blots

FIGURE 3C.

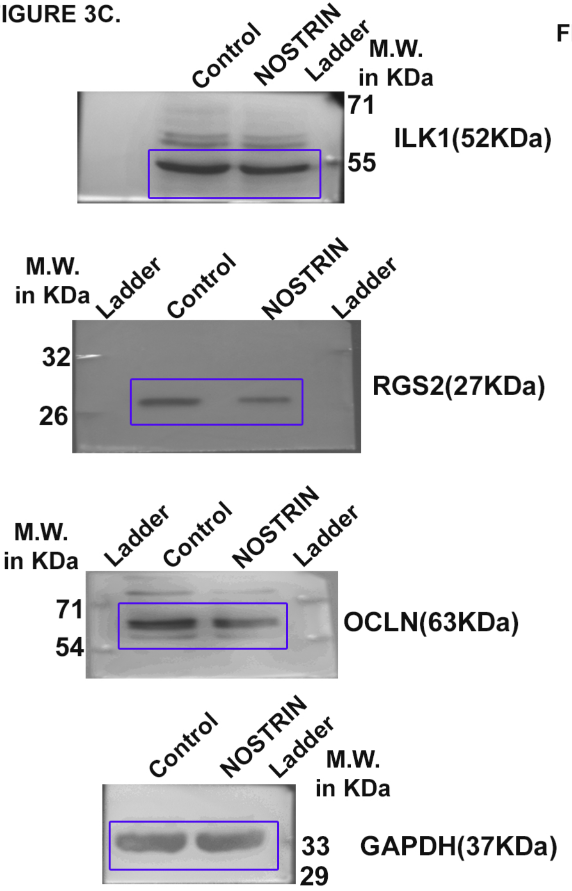

Cropped blots

FIGURE 3C.

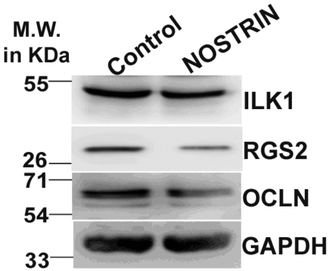

Original blots

FIGURE 3D.

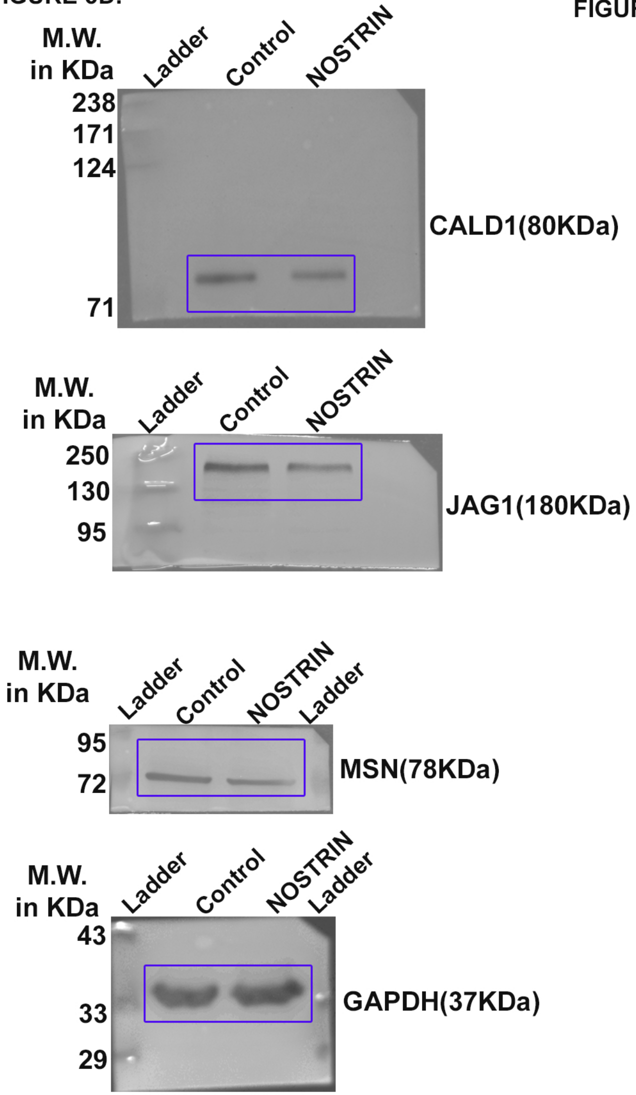

Cropped blots

FIGURE 3D.

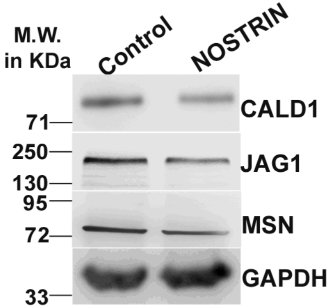

Original blots

FIGURE 3E.

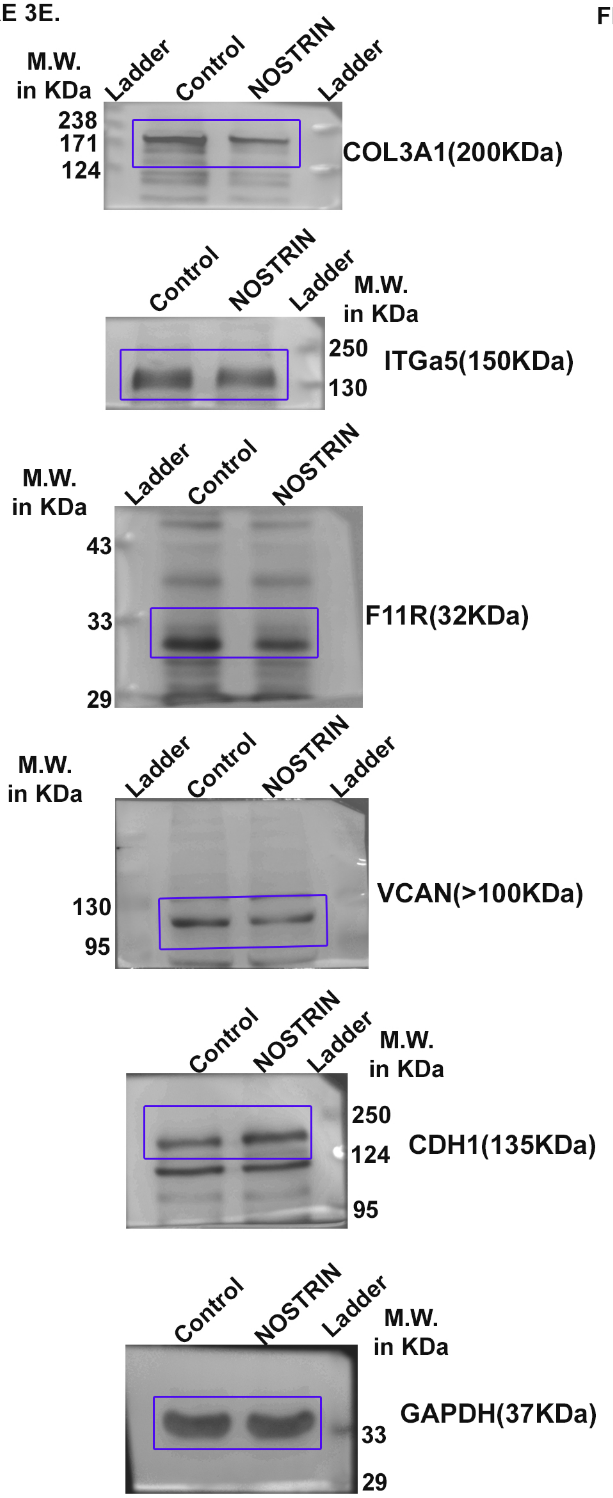

Cropped blots

FIGURE 3E.

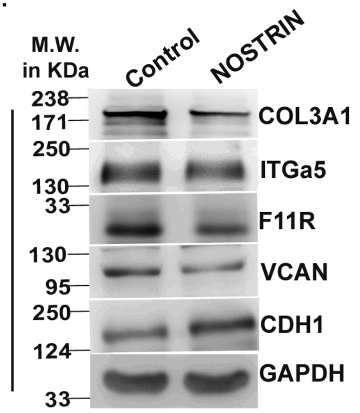

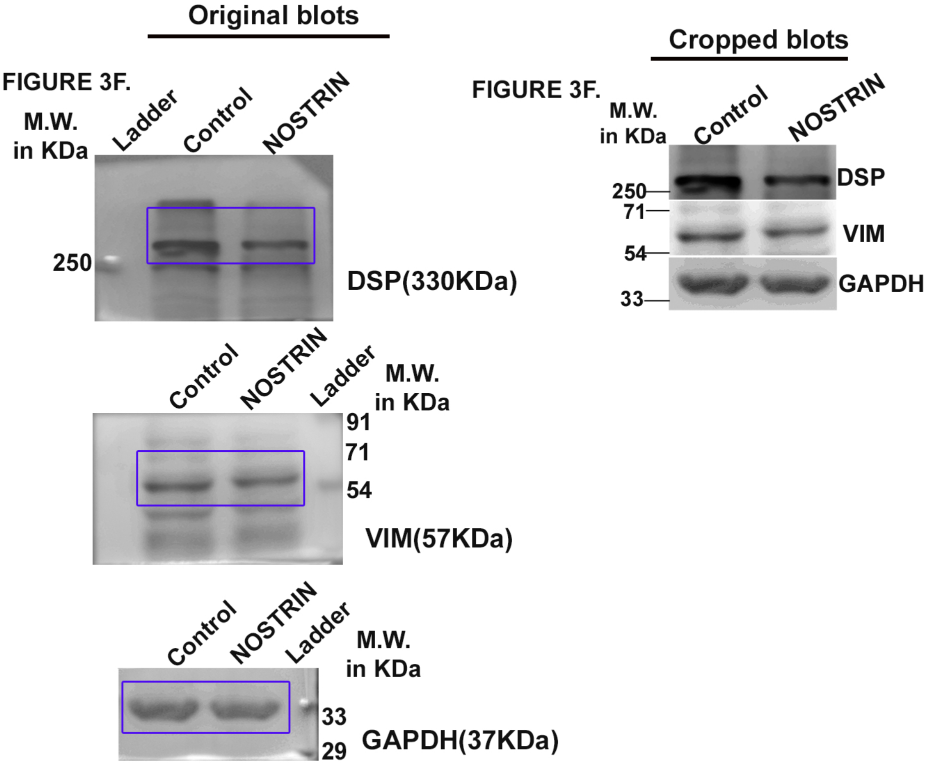

Original blots
Cropped blots
FIGURE 3F.
M.W. in KDa
Ladder
Control
NOSTRIN
250
DSP(330KDa)
91
71
54
VIM(57KDa)
33
29
GAPDH(37KDa)
DSP
VIM
GAPDH

Original blots

FIGURE 5H.

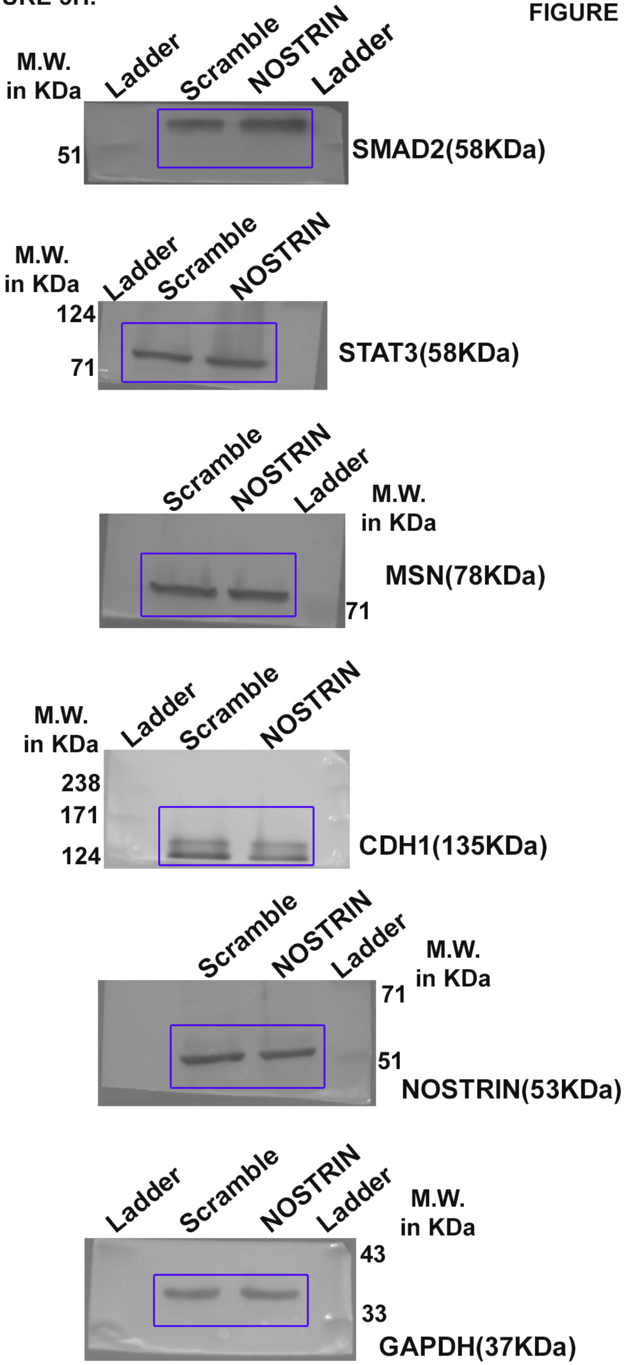

Cropped blots

FIGURE 5H.

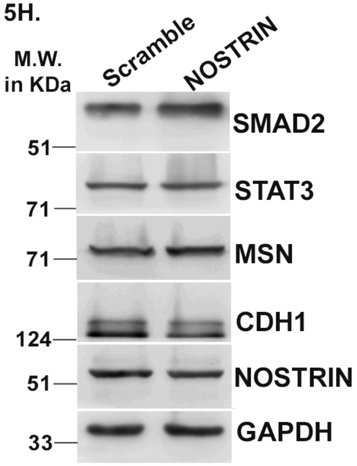

Original blots

Cropped blots

FIGURE 6C.

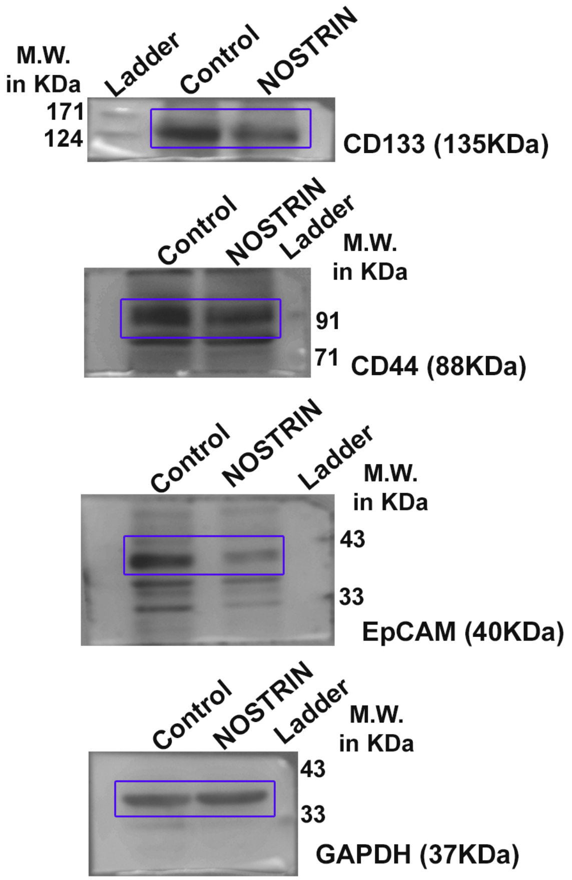

FIGURE 6C.

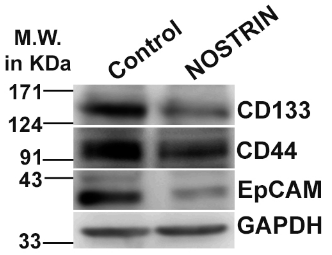

FIGURE 6F.

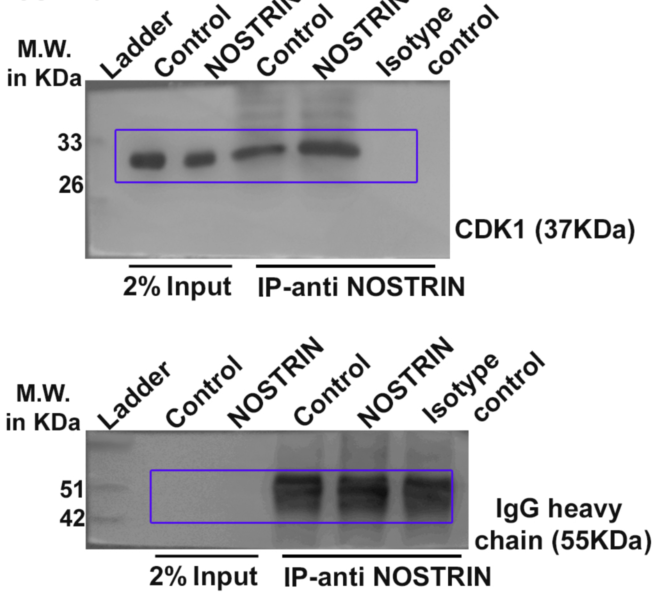

FIGURE 6F.

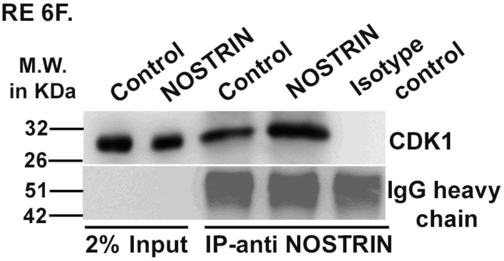

Original blots

FIGURE 6G.

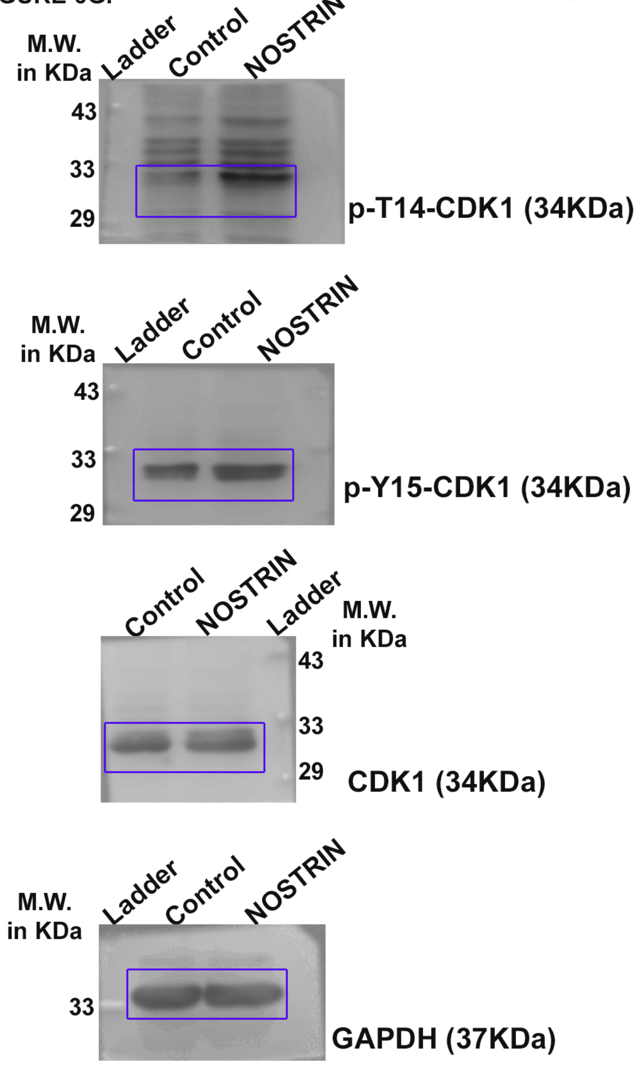

Cropped blots

FIGURE 6G.

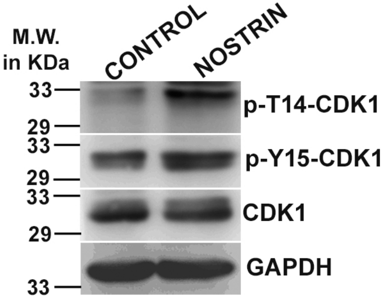

Original blots

FIGURE S1.

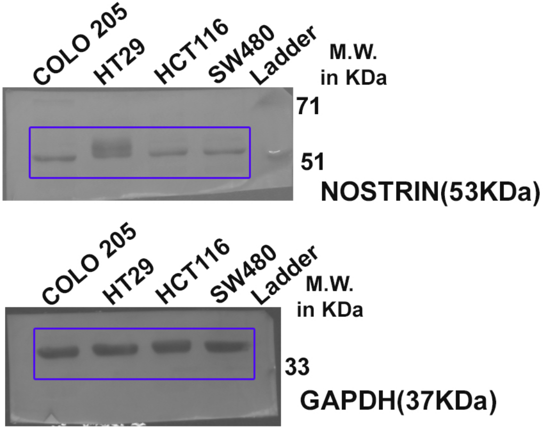

Cropped blots

FIGURE S1.

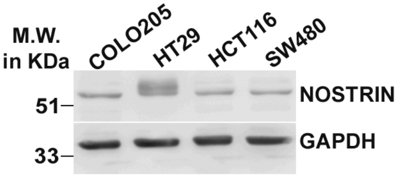

Supplement: Supplementary file 3 — Additional file 3: Fig. S3. Original full-length blots are compiled and shown along with their corresponding cropped versions. [file 12885_2022_9670_MOESM3_ESM.pdf]
